# Jet and underlying event properties as a function of charged-particle multiplicity in proton–proton collisions at $\sqrt {s}= 7\ \text{TeV}$

**DOI:** 10.1140/epjc/s10052-013-2674-5

**Published:** 2013-12-11

**Authors:** The CMS Collaboration, S. Chatrchyan, V. Khachatryan, A. M. Sirunyan, A. Tumasyan, W. Adam, T. Bergauer, M. Dragicevic, J. Erö, C. Fabjan, M. Friedl, R. Frühwirth, V. M. Ghete, N. Hörmann, J. Hrubec, M. Jeitler, W. Kiesenhofer, V. Knünz, M. Krammer, I. Krätschmer, D. Liko, I. Mikulec, D. Rabady, B. Rahbaran, C. Rohringer, H. Rohringer, R. Schöfbeck, J. Strauss, A. Taurok, W. Treberer-Treberspurg, W. Waltenberger, C.-E. Wulz, V. Mossolov, N. Shumeiko, J. Suarez Gonzalez, S. Alderweireldt, M. Bansal, S. Bansal, T. Cornelis, E. A. De Wolf, X. Janssen, A. Knutsson, S. Luyckx, L. Mucibello, S. Ochesanu, B. Roland, R. Rougny, Z. Staykova, H. Van Haevermaet, P. Van Mechelen, N. Van Remortel, A. Van Spilbeeck, F. Blekman, S. Blyweert, J. D’Hondt, A. Kalogeropoulos, J. Keaveney, S. Lowette, M. Maes, A. Olbrechts, S. Tavernier, W. Van Doninck, P. Van Mulders, G. P. Van Onsem, I. Villella, C. Caillol, B. Clerbaux, G. De Lentdecker, L. Favart, A. P. R. Gay, T. Hreus, A. Léonard, P. E. Marage, A. Mohammadi, L. Perniè, T. Reis, T. Seva, L. Thomas, C. Vander Velde, P. Vanlaer, J. Wang, V. Adler, K. Beernaert, L. Benucci, A. Cimmino, S. Costantini, S. Dildick, G. Garcia, B. Klein, J. Lellouch, A. Marinov, J. Mccartin, A. A. Ocampo Rios, D. Ryckbosch, M. Sigamani, N. Strobbe, F. Thyssen, M. Tytgat, S. Walsh, E. Yazgan, N. Zaganidis, S. Basegmez, C. Beluffi, G. Bruno, R. Castello, A. Caudron, L. Ceard, G. G. Da Silveira, C. Delaere, T. du Pree, D. Favart, L. Forthomme, A. Giammanco, J. Hollar, P. Jez, V. Lemaitre, J. Liao, O. Militaru, C. Nuttens, D. Pagano, A. Pin, K. Piotrzkowski, A. Popov, M. Selvaggi, M. Vidal Marono, J. M. Vizan Garcia, N. Beliy, T. Caebergs, E. Daubie, G. H. Hammad, G. A. Alves, M. Correa Martins Junior, T. Martins, M. E. Pol, M. H. G. Souza, W. L. Aldá Júnior, W. Carvalho, J. Chinellato, A. Custódio, E. M. Da Costa, D. De Jesus Damiao, C. De Oliveira Martins, S. Fonseca De Souza, H. Malbouisson, M. Malek, D. Matos Figueiredo, L. Mundim, H. Nogima, W. L. Prado Da Silva, A. Santoro, A. Sznajder, E. J. Tonelli Manganote, A. Vilela Pereira, C. A. Bernardes, F. A. Dias, T. R. Fernandez Perez Tomei, E. M. Gregores, C. Lagana, P. G. Mercadante, S. F. Novaes, Sandra S. Padula, V. Genchev, P. Iaydjiev, S. Piperov, M. Rodozov, G. Sultanov, M. Vutova, A. Dimitrov, R. Hadjiiska, V. Kozhuharov, L. Litov, B. Pavlov, P. Petkov, J. G. Bian, G. M. Chen, H. S. Chen, C. H. Jiang, D. Liang, S. Liang, X. Meng, J. Tao, X. Wang, Z. Wang, C. Asawatangtrakuldee, Y. Ban, Y. Guo, Q. Li, W. Li, S. Liu, Y. Mao, S. J. Qian, D. Wang, L. Zhang, W. Zou, C. Avila, C. A. Carrillo Montoya, L. F. Chaparro Sierra, J. P. Gomez, B. Gomez Moreno, J. C. Sanabria, N. Godinovic, D. Lelas, R. Plestina, D. Polic, I. Puljak, Z. Antunovic, M. Kovac, V. Brigljevic, K. Kadija, J. Luetic, D. Mekterovic, S. Morovic, L. Tikvica, A. Attikis, G. Mavromanolakis, J. Mousa, C. Nicolaou, F. Ptochos, P. A. Razis, M. Finger, M. Finger, A. A. Abdelalim, Y. Assran, S. Elgammal, A. Ellithi Kamel, M. A. Mahmoud, A. Radi, M. Kadastik, M. Müntel, M. Murumaa, M. Raidal, L. Rebane, A. Tiko, P. Eerola, G. Fedi, M. Voutilainen, J. Härkönen, V. Karimäki, R. Kinnunen, M. J. Kortelainen, T. Lampén, K. Lassila-Perini, S. Lehti, T. Lindén, P. Luukka, T. Mäenpää, T. Peltola, E. Tuominen, J. Tuominiemi, E. Tuovinen, L. Wendland, T. Tuuva, M. Besancon, F. Couderc, M. Dejardin, D. Denegri, B. Fabbro, J. L. Faure, F. Ferri, S. Ganjour, A. Givernaud, P. Gras, G. Hamel de Monchenault, P. Jarry, E. Locci, J. Malcles, L. Millischer, A. Nayak, J. Rander, A. Rosowsky, M. Titov, S. Baffioni, F. Beaudette, L. Benhabib, M. Bluj, P. Busson, C. Charlot, N. Daci, T. Dahms, M. Dalchenko, L. Dobrzynski, A. Florent, R. Granier de Cassagnac, M. Haguenauer, P. Miné, C. Mironov, I. N. Naranjo, M. Nguyen, C. Ochando, P. Paganini, D. Sabes, R. Salerno, Y. Sirois, C. Veelken, A. Zabi, J.-L. Agram, J. Andrea, D. Bloch, J.-M. Brom, E. C. Chabert, C. Collard, E. Conte, F. Drouhin, J.-C. Fontaine, D. Gelé, U. Goerlach, C. Goetzmann, P. Juillot, A.-C. Le Bihan, P. Van Hove, S. Gadrat, S. Beauceron, N. Beaupere, G. Boudoul, S. Brochet, J. Chasserat, R. Chierici, D. Contardo, P. Depasse, H. El Mamouni, J. Fan, J. Fay, S. Gascon, M. Gouzevitch, B. Ille, T. Kurca, M. Lethuillier, L. Mirabito, S. Perries, L. Sgandurra, V. Sordini, M. Vander Donckt, P. Verdier, S. Viret, H. Xiao, Z. Tsamalaidze, C. Autermann, S. Beranek, M. Bontenackels, B. Calpas, M. Edelhoff, L. Feld, N. Heracleous, O. Hindrichs, K. Klein, A. Ostapchuk, A. Perieanu, F. Raupach, J. Sammet, S. Schael, D. Sprenger, H. Weber, B. Wittmer, V. Zhukov, M. Ata, J. Caudron, E. Dietz-Laursonn, D. Duchardt, M. Erdmann, R. Fischer, A. Güth, T. Hebbeker, C. Heidemann, K. Hoepfner, D. Klingebiel, S. Knutzen, P. Kreuzer, M. Merschmeyer, A. Meyer, M. Olschewski, K. Padeken, P. Papacz, H. Pieta, H. Reithler, S. A. Schmitz, L. Sonnenschein, J. Steggemann, D. Teyssier, S. Thüer, M. Weber, V. Cherepanov, Y. Erdogan, G. Flügge, H. Geenen, M. Geisler, W. Haj Ahmad, F. Hoehle, B. Kargoll, T. Kress, Y. Kuessel, J. Lingemann, A. Nowack, I. M. Nugent, L. Perchalla, O. Pooth, A. Stahl, I. Asin, N. Bartosik, J. Behr, W. Behrenhoff, U. Behrens, A. J. Bell, M. Bergholz, A. Bethani, K. Borras, A. Burgmeier, A. Cakir, L. Calligaris, A. Campbell, S. Choudhury, F. Costanza, C. Diez Pardos, S. Dooling, T. Dorland, G. Eckerlin, D. Eckstein, G. Flucke, A. Geiser, I. Glushkov, A. Grebenyuk, P. Gunnellini, S. Habib, J. Hauk, G. Hellwig, D. Horton, H. Jung, M. Kasemann, P. Katsas, C. Kleinwort, H. Kluge, M. Krämer, D. Krücker, E. Kuznetsova, W. Lange, J. Leonard, K. Lipka, W. Lohmann, B. Lutz, R. Mankel, I. Marfin, I.-A. Melzer-Pellmann, A. B. Meyer, J. Mnich, A. Mussgiller, S. Naumann-Emme, O. Novgorodova, F. Nowak, J. Olzem, H. Perrey, A. Petrukhin, D. Pitzl, R. Placakyte, A. Raspereza, P. M. Ribeiro Cipriano, C. Riedl, E. Ron, M. Ö. Sahin, J. Salfeld-Nebgen, R. Schmidt, T. Schoerner-Sadenius, N. Sen, M. Stein, R. Walsh, C. Wissing, M. Aldaya Martin, V. Blobel, H. Enderle, J. Erfle, E. Garutti, U. Gebbert, M. Görner, M. Gosselink, J. Haller, K. Heine, R. S. Höing, G. Kaussen, H. Kirschenmann, R. Klanner, R. Kogler, J. Lange, I. Marchesini, T. Peiffer, N. Pietsch, D. Rathjens, C. Sander, H. Schettler, P. Schleper, E. Schlieckau, A. Schmidt, M. Schröder, T. Schum, M. Seidel, J. Sibille, V. Sola, H. Stadie, G. Steinbrück, J. Thomsen, D. Troendle, E. Usai, L. Vanelderen, C. Barth, C. Baus, J. Berger, C. Böser, E. Butz, T. Chwalek, W. De Boer, A. Descroix, A. Dierlamm, M. Feindt, M. Guthoff, F. Hartmann, T. Hauth, H. Held, K. H. Hoffmann, U. Husemann, I. Katkov, J. R. Komaragiri, A. Kornmayer, P. Lobelle Pardo, D. Martschei, M. U. Mozer, Th. Müller, M. Niegel, A. Nürnberg, O. Oberst, J. Ott, G. Quast, K. Rabbertz, F. Ratnikov, S. Röcker, F.-P. Schilling, G. Schott, H. J. Simonis, F. M. Stober, R. Ulrich, J. Wagner-Kuhr, S. Wayand, T. Weiler, M. Zeise, G. Anagnostou, G. Daskalakis, T. Geralis, S. Kesisoglou, A. Kyriakis, D. Loukas, A. Markou, C. Markou, E. Ntomari, I. Topsis-giotis, L. Gouskos, A. Panagiotou, N. Saoulidou, E. Stiliaris, X. Aslanoglou, I. Evangelou, G. Flouris, C. Foudas, P. Kokkas, N. Manthos, I. Papadopoulos, E. Paradas, G. Bencze, C. Hajdu, P. Hidas, D. Horvath, F. Sikler, V. Veszpremi, G. Vesztergombi, A. J. Zsigmond, N. Beni, S. Czellar, J. Molnar, J. Palinkas, Z. Szillasi, J. Karancsi, P. Raics, Z. L. Trocsanyi, B. Ujvari, S. K. Swain, S. B. Beri, V. Bhatnagar, N. Dhingra, R. Gupta, M. Kaur, M. Z. Mehta, M. Mittal, N. Nishu, A. Sharma, J. B. Singh, Ashok Kumar, Arun Kumar, S. Ahuja, A. Bhardwaj, B. C. Choudhary, A. Kumar, S. Malhotra, M. Naimuddin, K. Ranjan, P. Saxena, V. Sharma, R. K. Shivpuri, S. Banerjee, S. Bhattacharya, K. Chatterjee, S. Dutta, B. Gomber, Sa. Jain, Sh. Jain, R. Khurana, A. Modak, S. Mukherjee, D. Roy, S. Sarkar, M. Sharan, A. P. Singh, A. Abdulsalam, D. Dutta, S. Kailas, V. Kumar, A. K. Mohanty, L. M. Pant, P. Shukla, A. Topkar, T. Aziz, R. M. Chatterjee, S. Ganguly, S. Ghosh, M. Guchait, A. Gurtu, G. Kole, S. Kumar, M. Maity, G. Majumder, K. Mazumdar, G. B. Mohanty, B. Parida, K. Sudhakar, N. Wickramage, S. Banerjee, S. Dugad, H. Arfaei, H. Bakhshiansohi, S. M. Etesami, A. Fahim, A. Jafari, M. Khakzad, M. Mohammadi Najafabadi, S. Paktinat Mehdiabadi, B. Safarzadeh, M. Zeinali, M. Grunewald, M. Abbrescia, L. Barbone, C. Calabria, S. S. Chhibra, A. Colaleo, D. Creanza, N. De Filippis, M. De Palma, L. Fiore, G. Iaselli, G. Maggi, M. Maggi, B. Marangelli, S. My, S. Nuzzo, N. Pacifico, A. Pompili, G. Pugliese, G. Selvaggi, L. Silvestris, G. Singh, R. Venditti, P. Verwilligen, G. Zito, G. Abbiendi, A. C. Benvenuti, D. Bonacorsi, S. Braibant-Giacomelli, L. Brigliadori, R. Campanini, P. Capiluppi, A. Castro, F. R. Cavallo, G. Codispoti, M. Cuffiani, G. M. Dallavalle, F. Fabbri, A. Fanfani, D. Fasanella, P. Giacomelli, C. Grandi, L. Guiducci, S. Marcellini, G. Masetti, M. Meneghelli, A. Montanari, F. L. Navarria, F. Odorici, A. Perrotta, F. Primavera, A. M. Rossi, T. Rovelli, G. P. Siroli, N. Tosi, R. Travaglini, S. Albergo, M. Chiorboli, S. Costa, F. Giordano, R. Potenza, A. Tricomi, C. Tuve, G. Barbagli, V. Ciulli, C. Civinini, R. D’Alessandro, E. Focardi, S. Frosali, E. Gallo, S. Gonzi, V. Gori, P. Lenzi, M. Meschini, S. Paoletti, G. Sguazzoni, A. Tropiano, L. Benussi, S. Bianco, F. Fabbri, D. Piccolo, P. Fabbricatore, R. Ferretti, F. Ferro, M. Lo Vetere, R. Musenich, E. Robutti, S. Tosi, A. Benaglia, M. E. Dinardo, S. Fiorendi, S. Gennai, A. Ghezzi, P. Govoni, M. T. Lucchini, S. Malvezzi, R. A. Manzoni, A. Martelli, D. Menasce, L. Moroni, M. Paganoni, D. Pedrini, S. Ragazzi, N. Redaelli, T. Tabarelli de Fatis, S. Buontempo, N. Cavallo, A. De Cosa, F. Fabozzi, A. O. M. Iorio, L. Lista, S. Meola, M. Merola, P. Paolucci, P. Azzi, N. Bacchetta, M. Bellato, D. Bisello, A. Branca, R. Carlin, P. Checchia, T. Dorigo, F. Fanzago, M. Galanti, F. Gasparini, U. Gasparini, P. Giubilato, A. Gozzelino, K. Kanishchev, S. Lacaprara, I. Lazzizzera, M. Margoni, A. T. Meneguzzo, M. Passaseo, J. Pazzini, M. Pegoraro, N. Pozzobon, P. Ronchese, F. Simonetto, E. Torassa, M. Tosi, S. Vanini, P. Zotto, A. Zucchetta, G. Zumerle, M. Gabusi, S. P. Ratti, C. Riccardi, P. Vitulo, M. Biasini, G. M. Bilei, L. Fanò, P. Lariccia, G. Mantovani, M. Menichelli, A. Nappi, F. Romeo, A. Saha, A. Santocchia, A. Spiezia, K. Androsov, P. Azzurri, G. Bagliesi, J. Bernardini, T. Boccali, G. Broccolo, R. Castaldi, M. A. Ciocci, R. T. D’Agnolo, R. Dell’Orso, F. Fiori, L. Foà, A. Giassi, M. T. Grippo, A. Kraan, F. Ligabue, T. Lomtadze, L. Martini, A. Messineo, C. S. Moon, F. Palla, A. Rizzi, A. Savoy-Navarro, A. T. Serban, P. Spagnolo, P. Squillacioti, R. Tenchini, G. Tonelli, A. Venturi, P. G. Verdini, C. Vernieri, L. Barone, F. Cavallari, D. Del Re, M. Diemoz, M. Grassi, E. Longo, F. Margaroli, P. Meridiani, F. Micheli, S. Nourbakhsh, G. Organtini, R. Paramatti, S. Rahatlou, C. Rovelli, L. Soffi, N. Amapane, R. Arcidiacono, S. Argiro, M. Arneodo, R. Bellan, C. Biino, N. Cartiglia, S. Casasso, M. Costa, A. Degano, N. Demaria, C. Mariotti, S. Maselli, E. Migliore, V. Monaco, M. Musich, M. M. Obertino, N. Pastrone, M. Pelliccioni, A. Potenza, A. Romero, M. Ruspa, R. Sacchi, A. Solano, A. Staiano, U. Tamponi, S. Belforte, V. Candelise, M. Casarsa, F. Cossutti, G. Della Ricca, B. Gobbo, C. La Licata, M. Marone, D. Montanino, A. Penzo, A. Schizzi, A. Zanetti, S. Chang, T. Y. Kim, S. K. Nam, D. H. Kim, G. N. Kim, J. E. Kim, D. J. Kong, S. Lee, Y. D. Oh, H. Park, D. C. Son, J. Y. Kim, Zero J. Kim, S. Song, S. Choi, D. Gyun, B. Hong, M. Jo, H. Kim, T. J. Kim, K. S. Lee, S. K. Park, Y. Roh, M. Choi, J. H. Kim, C. Park, I. C. Park, S. Park, G. Ryu, Y. Choi, Y. K. Choi, J. Goh, M. S. Kim, E. Kwon, B. Lee, J. Lee, S. Lee, H. Seo, I. Yu, I. Grigelionis, A. Juodagalvis, H. Castilla-Valdez, E. De La Cruz-Burelo, I. Heredia-de La Cruz, R. Lopez-Fernandez, J. Martínez-Ortega, A. Sanchez-Hernandez, L. M. Villasenor-Cendejas, S. Carrillo Moreno, F. Vazquez Valencia, H. A. Salazar Ibarguen, E. Casimiro Linares, A. Morelos Pineda, M. A. Reyes-Santos, D. Krofcheck, P. H. Butler, R. Doesburg, S. Reucroft, H. Silverwood, M. Ahmad, M. I. Asghar, J. Butt, H. R. Hoorani, S. Khalid, W. A. Khan, T. Khurshid, S. Qazi, M. A. Shah, M. Shoaib, H. Bialkowska, B. Boimska, T. Frueboes, M. Górski, M. Kazana, K. Nawrocki, K. Romanowska-Rybinska, M. Szleper, G. Wrochna, P. Zalewski, G. Brona, K. Bunkowski, M. Cwiok, W. Dominik, K. Doroba, A. Kalinowski, M. Konecki, J. Krolikowski, M. Misiura, W. Wolszczak, N. Almeida, P. Bargassa, C. Beirão Da Cruz E Silva, P. Faccioli, P. G. Ferreira Parracho, M. Gallinaro, F. Nguyen, J. Rodrigues Antunes, J. Seixas, J. Varela, P. Vischia, S. Afanasiev, P. Bunin, M. Gavrilenko, I. Golutvin, I. Gorbunov, A. Kamenev, V. Karjavin, V. Konoplyanikov, A. Lanev, A. Malakhov, V. Matveev, P. Moisenz, V. Palichik, V. Perelygin, S. Shmatov, N. Skatchkov, V. Smirnov, A. Zarubin, S. Evstyukhin, V. Golovtsov, Y. Ivanov, V. Kim, P. Levchenko, V. Murzin, V. Oreshkin, I. Smirnov, V. Sulimov, L. Uvarov, S. Vavilov, A. Vorobyev, An. Vorobyev, Yu. Andreev, A. Dermenev, S. Gninenko, N. Golubev, M. Kirsanov, N. Krasnikov, A. Pashenkov, D. Tlisov, A. Toropin, V. Epshteyn, M. Erofeeva, V. Gavrilov, N. Lychkovskaya, V. Popov, G. Safronov, S. Semenov, A. Spiridonov, V. Stolin, E. Vlasov, A. Zhokin, V. Andreev, M. Azarkin, I. Dremin, M. Kirakosyan, A. Leonidov, G. Mesyats, S. V. Rusakov, A. Vinogradov, A. Belyaev, E. Boos, L. Dudko, A. Gribushin, L. Khein, V. Klyukhin, O. Kodolova, I. Lokhtin, A. Markina, S. Obraztsov, S. Petrushanko, A. Proskuryakov, V. Savrin, A. Snigirev, I. Azhgirey, I. Bayshev, S. Bitioukov, V. Kachanov, A. Kalinin, D. Konstantinov, V. Krychkine, V. Petrov, R. Ryutin, A. Sobol, L. Tourtchanovitch, S. Troshin, N. Tyurin, A. Uzunian, A. Volkov, P. Adzic, M. Djordjevic, M. Ekmedzic, D. Krpic, J. Milosevic, M. Aguilar-Benitez, J. Alcaraz Maestre, C. Battilana, E. Calvo, M. Cerrada, M. Chamizo Llatas, N. Colino, B. De La Cruz, A. Delgado Peris, D. Domínguez Vázquez, C. Fernandez Bedoya, J. P. Fernández Ramos, A. Ferrando, J. Flix, M. C. Fouz, P. Garcia-Abia, O. Gonzalez Lopez, S. Goy Lopez, J. M. Hernandez, M. I. Josa, G. Merino, E. Navarro De Martino, J. Puerta Pelayo, A. Quintario Olmeda, I. Redondo, L. Romero, J. Santaolalla, M. S. Soares, C. Willmott, C. Albajar, J. F. de Trocóniz, H. Brun, J. Cuevas, J. Fernandez Menendez, S. Folgueras, I. Gonzalez Caballero, L. Lloret Iglesias, J. Piedra Gomez, J. A. Brochero Cifuentes, I. J. Cabrillo, A. Calderon, S. H. Chuang, J. Duarte Campderros, M. Fernandez, G. Gomez, J. Gonzalez Sanchez, A. Graziano, C. Jorda, A. Lopez Virto, J. Marco, R. Marco, C. Martinez Rivero, F. Matorras, F. J. Munoz Sanchez, T. Rodrigo, A. Y. Rodríguez-Marrero, A. Ruiz-Jimeno, L. Scodellaro, I. Vila, R. Vilar Cortabitarte, D. Abbaneo, E. Auffray, G. Auzinger, M. Bachtis, P. Baillon, A. H. Ball, D. Barney, J. Bendavid, J. F. Benitez, C. Bernet, G. Bianchi, P. Bloch, A. Bocci, A. Bonato, O. Bondu, C. Botta, H. Breuker, T. Camporesi, G. Cerminara, T. Christiansen, J. A. Coarasa Perez, S. Colafranceschi, M. D’Alfonso, D. d’Enterria, A. Dabrowski, A. David, F. De Guio, A. De Roeck, S. De Visscher, S. Di Guida, M. Dobson, N. Dupont-Sagorin, A. Elliott-Peisert, J. Eugster, G. Franzoni, W. Funk, G. Georgiou, M. Giffels, D. Gigi, K. Gill, D. Giordano, M. Girone, M. Giunta, F. Glege, R. Gomez-Reino Garrido, S. Gowdy, R. Guida, J. Hammer, M. Hansen, P. Harris, C. Hartl, A. Hinzmann, V. Innocente, P. Janot, E. Karavakis, K. Kousouris, K. Krajczar, P. Lecoq, Y.-J. Lee, C. Lourenço, N. Magini, L. Malgeri, M. Mannelli, L. Masetti, F. Meijers, S. Mersi, E. Meschi, R. Moser, M. Mulders, P. Musella, E. Nesvold, L. Orsini, E. Palencia Cortezon, E. Perez, L. Perrozzi, A. Petrilli, A. Pfeiffer, M. Pierini, M. Pimiä, D. Piparo, M. Plagge, L. Quertenmont, A. Racz, W. Reece, G. Rolandi, M. Rovere, H. Sakulin, F. Santanastasio, C. Schäfer, C. Schwick, S. Sekmen, A. Sharma, P. Siegrist, P. Silva, M. Simon, P. Sphicas, D. Spiga, M. Stoye, A. Tsirou, G. I. Veres, J. R. Vlimant, H. K. Wöhri, S. D. Worm, W. D. Zeuner, W. Bertl, K. Deiters, W. Erdmann, K. Gabathuler, R. Horisberger, Q. Ingram, H. C. Kaestli, S. König, D. Kotlinski, U. Langenegger, D. Renker, T. Rohe, F. Bachmair, L. Bäni, L. Bianchini, P. Bortignon, M. A. Buchmann, B. Casal, N. Chanon, A. Deisher, G. Dissertori, M. Dittmar, M. Donegà, M. Dünser, P. Eller, K. Freudenreich, C. Grab, D. Hits, P. Lecomte, W. Lustermann, B. Mangano, A. C. Marini, P. Martinez Ruiz del Arbol, D. Meister, N. Mohr, F. Moortgat, C. Nägeli, P. Nef, F. Nessi-Tedaldi, F. Pandolfi, L. Pape, F. Pauss, M. Peruzzi, M. Quittnat, F. J. Ronga, M. Rossini, L. Sala, A. K. Sanchez, A. Starodumov, B. Stieger, M. Takahashi, L. Tauscher, A. Thea, K. Theofilatos, D. Treille, C. Urscheler, R. Wallny, H. A. Weber, C. Amsler, V. Chiochia, C. Favaro, M. Ivova Rikova, B. Kilminster, B. Millan Mejias, P. Robmann, H. Snoek, S. Taroni, M. Verzetti, Y. Yang, M. Cardaci, K. H. Chen, C. Ferro, C. M. Kuo, S. W. Li, W. Lin, Y. J. Lu, R. Volpe, S. S. Yu, P. Bartalini, P. Chang, Y. H. Chang, Y. W. Chang, Y. Chao, K. F. Chen, C. Dietz, U. Grundler, W.-S. Hou, Y. Hsiung, K. Y. Kao, Y. J. Lei, R.-S. Lu, D. Majumder, E. Petrakou, X. Shi, J. G. Shiu, Y. M. Tzeng, M. Wang, B. Asavapibhop, N. Suwonjandee, A. Adiguzel, M. N. Bakirci, S. Cerci, C. Dozen, I. Dumanoglu, E. Eskut, S. Girgis, G. Gokbulut, E. Gurpinar, I. Hos, E. E. Kangal, A. Kayis Topaksu, G. Onengut, K. Ozdemir, S. Ozturk, A. Polatoz, K. Sogut, D. Sunar Cerci, B. Tali, H. Topakli, M. Vergili, I. V. Akin, T. Aliev, B. Bilin, S. Bilmis, M. Deniz, H. Gamsizkan, A. M. Guler, G. Karapinar, K. Ocalan, A. Ozpineci, M. Serin, R. Sever, U. E. Surat, M. Yalvac, M. Zeyrek, E. Gülmez, B. Isildak, M. Kaya, O. Kaya, S. Ozkorucuklu, N. Sonmez, H. Bahtiyar, E. Barlas, K. Cankocak, Y. O. Günaydin, F. I. Vardarlı, M. Yücel, L. Levchuk, P. Sorokin, J. J. Brooke, E. Clement, D. Cussans, H. Flacher, R. Frazier, J. Goldstein, M. Grimes, G. P. Heath, H. F. Heath, L. Kreczko, C. Lucas, Z. Meng, S. Metson, D. M. Newbold, K. Nirunpong, S. Paramesvaran, A. Poll, S. Senkin, V. J. Smith, T. Williams, K. W. Bell, A. Belyaev, C. Brew, R. M. Brown, D. J. A. Cockerill, J. A. Coughlan, K. Harder, S. Harper, J. Ilic, E. Olaiya, D. Petyt, B. C. Radburn-Smith, C. H. Shepherd-Themistocleous, I. R. Tomalin, W. J. Womersley, R. Bainbridge, O. Buchmuller, D. Burton, D. Colling, N. Cripps, M. Cutajar, P. Dauncey, G. Davies, M. Della Negra, W. Ferguson, J. Fulcher, D. Futyan, A. Gilbert, A. Guneratne Bryer, G. Hall, Z. Hatherell, J. Hays, G. Iles, M. Jarvis, G. Karapostoli, M. Kenzie, R. Lane, R. Lucas, L. Lyons, A.-M. Magnan, J. Marrouche, B. Mathias, R. Nandi, J. Nash, A. Nikitenko, J. Pela, M. Pesaresi, K. Petridis, M. Pioppi, D. M. Raymond, S. Rogerson, A. Rose, C. Seez, P. Sharp, A. Sparrow, A. Tapper, M. Vazquez Acosta, T. Virdee, S. Wakefield, N. Wardle, M. Chadwick, J. E. Cole, P. R. Hobson, A. Khan, P. Kyberd, D. Leggat, D. Leslie, W. Martin, I. D. Reid, P. Symonds, L. Teodorescu, M. Turner, J. Dittmann, K. Hatakeyama, A. Kasmi, H. Liu, T. Scarborough, O. Charaf, S. I. Cooper, C. Henderson, P. Rumerio, A. Avetisyan, T. Bose, C. Fantasia, A. Heister, P. Lawson, D. Lazic, J. Rohlf, D. Sperka, J. St. John, L. Sulak, J. Alimena, S. Bhattacharya, G. Christopher, D. Cutts, Z. Demiragli, A. Ferapontov, A. Garabedian, U. Heintz, S. Jabeen, G. Kukartsev, E. Laird, G. Landsberg, M. Luk, M. Narain, M. Segala, T. Sinthuprasith, T. Speer, R. Breedon, G. Breto, M. Calderon De La Barca Sanchez, S. Chauhan, M. Chertok, J. Conway, R. Conway, P. T. Cox, R. Erbacher, M. Gardner, R. Houtz, W. Ko, A. Kopecky, R. Lander, T. Miceli, D. Pellett, J. Pilot, F. Ricci-Tam, B. Rutherford, M. Searle, J. Smith, M. Squires, M. Tripathi, S. Wilbur, R. Yohay, V. Andreev, D. Cline, R. Cousins, S. Erhan, P. Everaerts, C. Farrell, M. Felcini, J. Hauser, M. Ignatenko, C. Jarvis, G. Rakness, P. Schlein, E. Takasugi, P. Traczyk, V. Valuev, M. Weber, J. Babb, R. Clare, J. Ellison, J. W. Gary, G. Hanson, J. Heilman, P. Jandir, H. Liu, O. R. Long, A. Luthra, M. Malberti, H. Nguyen, A. Shrinivas, J. Sturdy, S. Sumowidagdo, R. Wilken, S. Wimpenny, W. Andrews, J. G. Branson, G. B. Cerati, S. Cittolin, D. Evans, A. Holzner, R. Kelley, M. Lebourgeois, J. Letts, I. Macneill, S. Padhi, C. Palmer, G. Petrucciani, M. Pieri, M. Sani, V. Sharma, S. Simon, E. Sudano, M. Tadel, Y. Tu, A. Vartak, S. Wasserbaech, F. Würthwein, A. Yagil, J. Yoo, D. Barge, C. Campagnari, T. Danielson, K. Flowers, P. Geffert, C. George, F. Golf, J. Incandela, C. Justus, D. Kovalskyi, V. Krutelyov, R. Magaña Villalba, N. Mccoll, V. Pavlunin, J. Richman, R. Rossin, D. Stuart, W. To, C. West, A. Apresyan, A. Bornheim, J. Bunn, Y. Chen, E. Di Marco, J. Duarte, D. Kcira, Y. Ma, A. Mott, H. B. Newman, C. Pena, C. Rogan, M. Spiropulu, V. Timciuc, J. Veverka, R. Wilkinson, S. Xie, R. Y. Zhu, V. Azzolini, A. Calamba, R. Carroll, T. Ferguson, Y. Iiyama, D. W. Jang, Y. F. Liu, M. Paulini, J. Russ, H. Vogel, I. Vorobiev, J. P. Cumalat, B. R. Drell, W. T. Ford, A. Gaz, E. Luiggi Lopez, U. Nauenberg, J. G. Smith, K. Stenson, K. A. Ulmer, S. R. Wagner, J. Alexander, A. Chatterjee, N. Eggert, L. K. Gibbons, W. Hopkins, A. Khukhunaishvili, B. Kreis, N. Mirman, G. Nicolas Kaufman, J. R. Patterson, A. Ryd, E. Salvati, W. Sun, W. D. Teo, J. Thom, J. Thompson, J. Tucker, Y. Weng, L. Winstrom, P. Wittich, D. Winn, S. Abdullin, M. Albrow, J. Anderson, G. Apollinari, L. A. T. Bauerdick, A. Beretvas, J. Berryhill, P. C. Bhat, K. Burkett, J. N. Butler, V. Chetluru, H. W. K. Cheung, F. Chlebana, S. Cihangir, V. D. Elvira, I. Fisk, J. Freeman, Y. Gao, E. Gottschalk, L. Gray, D. Green, O. Gutsche, D. Hare, R. M. Harris, J. Hirschauer, B. Hooberman, S. Jindariani, M. Johnson, U. Joshi, K. Kaadze, B. Klima, S. Kunori, S. Kwan, J. Linacre, D. Lincoln, R. Lipton, J. Lykken, K. Maeshima, J. M. Marraffino, V. I. Martinez Outschoorn, S. Maruyama, D. Mason, P. McBride, K. Mishra, S. Mrenna, Y. Musienko, C. Newman-Holmes, V. O’Dell, O. Prokofyev, N. Ratnikova, E. Sexton-Kennedy, S. Sharma, W. J. Spalding, L. Spiegel, L. Taylor, S. Tkaczyk, N. V. Tran, L. Uplegger, E. W. Vaandering, R. Vidal, J. Whitmore, W. Wu, F. Yang, J. C. Yun, D. Acosta, P. Avery, D. Bourilkov, M. Chen, T. Cheng, S. Das, M. De Gruttola, G. P. Di Giovanni, D. Dobur, A. Drozdetskiy, R. D. Field, M. Fisher, Y. Fu, I. K. Furic, J. Hugon, B. Kim, J. Konigsberg, A. Korytov, A. Kropivnitskaya, T. Kypreos, J. F. Low, K. Matchev, P. Milenovic, G. Mitselmakher, L. Muniz, R. Remington, A. Rinkevicius, N. Skhirtladze, M. Snowball, J. Yelton, M. Zakaria, V. Gaultney, S. Hewamanage, S. Linn, P. Markowitz, G. Martinez, J. L. Rodriguez, T. Adams, A. Askew, J. Bochenek, J. Chen, B. Diamond, J. Haas, S. Hagopian, V. Hagopian, K. F. Johnson, H. Prosper, V. Veeraraghavan, M. Weinberg, M. M. Baarmand, B. Dorney, M. Hohlmann, H. Kalakhety, F. Yumiceva, M. R. Adams, L. Apanasevich, V. E. Bazterra, R. R. Betts, I. Bucinskaite, J. Callner, R. Cavanaugh, O. Evdokimov, L. Gauthier, C. E. Gerber, D. J. Hofman, S. Khalatyan, P. Kurt, F. Lacroix, D. H. Moon, C. O’Brien, C. Silkworth, D. Strom, P. Turner, N. Varelas, U. Akgun, E. A. Albayrak, B. Bilki, W. Clarida, K. Dilsiz, F. Duru, S. Griffiths, J.-P. Merlo, H. Mermerkaya, A. Mestvirishvili, A. Moeller, J. Nachtman, C. R. Newsom, H. Ogul, Y. Onel, F. Ozok, S. Sen, P. Tan, E. Tiras, J. Wetzel, T. Yetkin, K. Yi, B. A. Barnett, B. Blumenfeld, S. Bolognesi, G. Giurgiu, A. V. Gritsan, G. Hu, P. Maksimovic, C. Martin, M. Swartz, A. Whitbeck, P. Baringer, A. Bean, G. Benelli, R. P. Kenny III, M. Murray, D. Noonan, S. Sanders, R. Stringer, J. S. Wood, A. F. Barfuss, I. Chakaberia, A. Ivanov, S. Khalil, M. Makouski, Y. Maravin, L. K. Saini, S. Shrestha, I. Svintradze, J. Gronberg, D. Lange, F. Rebassoo, D. Wright, A. Baden, B. Calvert, S. C. Eno, J. A. Gomez, N. J. Hadley, R. G. Kellogg, T. Kolberg, Y. Lu, M. Marionneau, A. C. Mignerey, K. Pedro, A. Peterman, A. Skuja, J. Temple, M. B. Tonjes, S. C. Tonwar, A. Apyan, G. Bauer, W. Busza, I. A. Cali, M. Chan, L. Di Matteo, V. Dutta, G. Gomez Ceballos, M. Goncharov, D. Gulhan, Y. Kim, M. Klute, Y. S. Lai, A. Levin, P. D. Luckey, T. Ma, S. Nahn, C. Paus, D. Ralph, C. Roland, G. Roland, G. S. F. Stephans, F. Stöckli, K. Sumorok, D. Velicanu, R. Wolf, B. Wyslouch, M. Yang, Y. Yilmaz, A. S. Yoon, M. Zanetti, V. Zhukova, B. Dahmes, A. De Benedetti, A. Gude, J. Haupt, S. C. Kao, K. Klapoetke, Y. Kubota, J. Mans, N. Pastika, R. Rusack, M. Sasseville, A. Singovsky, N. Tambe, J. Turkewitz, J. G. Acosta, L. M. Cremaldi, R. Kroeger, S. Oliveros, L. Perera, R. Rahmat, D. A. Sanders, D. Summers, E. Avdeeva, K. Bloom, S. Bose, D. R. Claes, A. Dominguez, M. Eads, R. Gonzalez Suarez, J. Keller, I. Kravchenko, J. Lazo-Flores, S. Malik, F. Meier, G. R. Snow, J. Dolen, A. Godshalk, I. Iashvili, S. Jain, A. Kharchilava, A. Kumar, S. Rappoccio, Z. Wan, G. Alverson, E. Barberis, D. Baumgartel, M. Chasco, J. Haley, A. Massironi, D. Nash, T. Orimoto, D. Trocino, D. Wood, J. Zhang, A. Anastassov, K. A. Hahn, A. Kubik, L. Lusito, N. Mucia, N. Odell, B. Pollack, A. Pozdnyakov, M. Schmitt, S. Stoynev, K. Sung, M. Velasco, S. Won, D. Berry, A. Brinkerhoff, K. M. Chan, M. Hildreth, C. Jessop, D. J. Karmgard, J. Kolb, K. Lannon, W. Luo, S. Lynch, N. Marinelli, D. M. Morse, T. Pearson, M. Planer, R. Ruchti, J. Slaunwhite, N. Valls, M. Wayne, M. Wolf, L. Antonelli, B. Bylsma, L. S. Durkin, C. Hill, R. Hughes, K. Kotov, T. Y. Ling, D. Puigh, M. Rodenburg, G. Smith, C. Vuosalo, B. L. Winer, H. Wolfe, E. Berry, P. Elmer, V. Halyo, P. Hebda, J. Hegeman, A. Hunt, P. Jindal, S. A. Koay, P. Lujan, D. Marlow, T. Medvedeva, M. Mooney, J. Olsen, P. Piroué, X. Quan, A. Raval, H. Saka, D. Stickland, C. Tully, J. S. Werner, S. C. Zenz, A. Zuranski, E. Brownson, A. Lopez, H. Mendez, J. E. Ramirez Vargas, E. Alagoz, D. Benedetti, G. Bolla, D. Bortoletto, M. De Mattia, A. Everett, Z. Hu, M. Jones, K. Jung, O. Koybasi, M. Kress, N. Leonardo, D. Lopes Pegna, V. Maroussov, P. Merkel, D. H. Miller, N. Neumeister, I. Shipsey, D. Silvers, A. Svyatkovskiy, F. Wang, W. Xie, L. Xu, H. D. Yoo, J. Zablocki, Y. Zheng, N. Parashar, A. Adair, B. Akgun, K. M. Ecklund, F. J. M. Geurts, W. Li, B. Michlin, B. P. Padley, R. Redjimi, J. Roberts, J. Zabel, B. Betchart, A. Bodek, R. Covarelli, P. de Barbaro, R. Demina, Y. Eshaq, T. Ferbel, A. Garcia-Bellido, P. Goldenzweig, J. Han, A. Harel, D. C. Miner, G. Petrillo, D. Vishnevskiy, M. Zielinski, A. Bhatti, R. Ciesielski, L. Demortier, K. Goulianos, G. Lungu, S. Malik, C. Mesropian, S. Arora, A. Barker, J. P. Chou, C. Contreras-Campana, E. Contreras-Campana, D. Duggan, D. Ferencek, Y. Gershtein, R. Gray, E. Halkiadakis, D. Hidas, A. Lath, S. Panwalkar, M. Park, R. Patel, V. Rekovic, J. Robles, S. Salur, S. Schnetzer, C. Seitz, S. Somalwar, R. Stone, S. Thomas, P. Thomassen, M. Walker, G. Cerizza, M. Hollingsworth, K. Rose, S. Spanier, Z. C. Yang, A. York, O. Bouhali, R. Eusebi, W. Flanagan, J. Gilmore, T. Kamon, V. Khotilovich, R. Montalvo, I. Osipenkov, Y. Pakhotin, A. Perloff, J. Roe, A. Safonov, T. Sakuma, I. Suarez, A. Tatarinov, D. Toback, N. Akchurin, C. Cowden, J. Damgov, C. Dragoiu, P. R. Dudero, K. Kovitanggoon, S. W. Lee, T. Libeiro, I. Volobouev, E. Appelt, A. G. Delannoy, S. Greene, A. Gurrola, W. Johns, C. Maguire, Y. Mao, A. Melo, M. Sharma, P. Sheldon, B. Snook, S. Tuo, J. Velkovska, M. W. Arenton, S. Boutle, B. Cox, B. Francis, J. Goodell, R. Hirosky, A. Ledovskoy, C. Lin, C. Neu, J. Wood, S. Gollapinni, R. Harr, P. E. Karchin, C. Kottachchi Kankanamge Don, P. Lamichhane, A. Sakharov, D. A. Belknap, L. Borrello, D. Carlsmith, M. Cepeda, S. Dasu, S. Duric, E. Friis, M. Grothe, R. Hall-Wilton, M. Herndon, A. Hervé, P. Klabbers, J. Klukas, A. Lanaro, R. Loveless, A. Mohapatra, I. Ojalvo, T. Perry, G. A. Pierro, G. Polese, I. Ross, T. Sarangi, A. Savin, W. H. Smith, J. Swanson

**Affiliations:** 1 CERN, Geneva, Switzerland; 2 Yerevan Physics Institute, Yerevan, Armenia; 3 Institut für Hochenergiephysik der OeAW, Wien, Austria; 4 National Centre for Particle and High Energy Physics, Minsk, Belarus; 5 Universiteit Antwerpen, Antwerpen, Belgium; 6 Vrije Universiteit Brussel, Brussel, Belgium; 7 Université Libre de Bruxelles, Bruxelles, Belgium; 8 Ghent University, Ghent, Belgium; 9 Université Catholique de Louvain, Louvain-la-Neuve, Belgium; 10 Université de Mons, Mons, Belgium; 11 Centro Brasileiro de Pesquisas Fisicas, Rio de Janeiro, Brazil; 12 Universidade do Estado do Rio de Janeiro, Rio de Janeiro, Brazil; 13 Universidade Estadual Paulista, São Paulo, Brazil; 14 Universidade Federal do ABC, São Paulo, Brazil; 15 Institute for Nuclear Research and Nuclear Energy, Sofia, Bulgaria; 16 University of Sofia, Sofia, Bulgaria; 17 Institute of High Energy Physics, Beijing, China; 18 State Key Laboratory of Nuclear Physics and Technology, Peking University, Beijing, China; 19 Universidad de Los Andes, Bogota, Colombia; 20 Technical University of Split, Split, Croatia; 21 University of Split, Split, Croatia; 22 Institute Rudjer Boskovic, Zagreb, Croatia; 23 University of Cyprus, Nicosia, Cyprus; 24 Charles University, Prague, Czech Republic; 25 Egyptian Network of High Energy Physics, Academy of Scientific Research and Technology of the Arab Republic of Egypt, Cairo, Egypt; 26 National Institute of Chemical Physics and Biophysics, Tallinn, Estonia; 27 Department of Physics, University of Helsinki, Helsinki, Finland; 28 Helsinki Institute of Physics, Helsinki, Finland; 29 Lappeenranta University of Technology, Lappeenranta, Finland; 30 DSM/IRFU, CEA/Saclay, Gif-sur-Yvette, France; 31 Laboratoire Leprince-Ringuet, Ecole Polytechnique, IN2P3-CNRS, Palaiseau, France; 32 Institut Pluridisciplinaire Hubert Curien, Université de Strasbourg, Université de Haute Alsace Mulhouse, CNRS/IN2P3, Strasbourg, France; 33 Centre de Calcul de l’Institut National de Physique Nucleaire et de Physique des Particules, CNRS/IN2P3, Villeurbanne, France; 34 CNRS-IN2P3, Institut de Physique Nucléaire de Lyon, Université de Lyon, Université Claude Bernard Lyon 1, Villeurbanne, France; 35 Institute of High Energy Physics and Informatization, Tbilisi State University, Tbilisi, Georgia; 36 I. Physikalisches Institut, RWTH Aachen University, Aachen, Germany; 37 III. Physikalisches Institut A, RWTH Aachen University, Aachen, Germany; 38 III. Physikalisches Institut B, RWTH Aachen University, Aachen, Germany; 39 Deutsches Elektronen-Synchrotron, Hamburg, Germany; 40 University of Hamburg, Hamburg, Germany; 41 Institut für Experimentelle Kernphysik, Karlsruhe, Germany; 42 Institute of Nuclear and Particle Physics (INPP), NCSR Demokritos, Aghia Paraskevi, Greece; 43 University of Athens, Athens, Greece; 44 University of Ioánnina, Ioánnina, Greece; 45 KFKI Research Institute for Particle and Nuclear Physics, Budapest, Hungary; 46 Institute of Nuclear Research ATOMKI, Debrecen, Hungary; 47 University of Debrecen, Debrecen, Hungary; 48 National Institute of Science Education and Research, Bhubaneswar, India; 49 Panjab University, Chandigarh, India; 50 University of Delhi, Delhi, India; 51 Saha Institute of Nuclear Physics, Kolkata, India; 52 Bhabha Atomic Research Centre, Mumbai, India; 53 Tata Institute of Fundamental Research - EHEP, Mumbai, India; 54 Tata Institute of Fundamental Research - HECR, Mumbai, India; 55 Institute for Research in Fundamental Sciences (IPM), Tehran, Iran; 56 University College Dublin, Dublin, Ireland; 57 INFN Sezione di Bari, Bari, Italy; 58 Università di Bari, Bari, Italy; 59 Politecnico di Bari, Bari, Italy; 60 INFN Sezione di Bologna, Bologna, Italy; 61 Università di Bologna, Bologna, Italy; 62 INFN Sezione di Catania, Catania, Italy; 63 Università di Catania, Catania, Italy; 64 INFN Sezione di Firenze, Firenze, Italy; 65 Università di Firenze, Firenze, Italy; 66 INFN Laboratori Nazionali di Frascati, Frascati, Italy; 67 INFN Sezione di Genova, Genova, Italy; 68 Università di Genova, Genova, Italy; 69 INFN Sezione di Milano-Bicocca, Milano, Italy; 70 Università di Milano-Bicocca, Milano, Italy; 71 INFN Sezione di Napoli, Napoli, Italy; 72 Università di Napoli ‘Federico II’, Napoli, Italy; 73 Università della Basilicata (Potenza), Napoli, Italy; 74 Università G. Marconi (Roma), Napoli, Italy; 75 INFN Sezione di Padova, Padova, Italy; 76 Università di Padova, Padova, Italy; 77 Università di Trento (Trento), Padova, Italy; 78 INFN Sezione di Pavia, Pavia, Italy; 79 Università di Pavia, Pavia, Italy; 80 INFN Sezione di Perugia, Perugia, Italy; 81 Università di Perugia, Perugia, Italy; 82 INFN Sezione di Pisa, Pisa, Italy; 83 Università di Pisa, Pisa, Italy; 84 Scuola Normale Superiore di Pisa, Pisa, Italy; 85 INFN Sezione di Roma, Roma, Italy; 86 Università di Roma, Roma, Italy; 87 INFN Sezione di Torino, Torino, Italy; 88 Università di Torino, Torino, Italy; 89 Università del Piemonte Orientale (Novara), Torino, Italy; 90 INFN Sezione di Trieste, Trieste, Italy; 91 Università di Trieste, Trieste, Italy; 92 Kangwon National University, Chunchon, Korea; 93 Kyungpook National University, Daegu, Korea; 94 Institute for Universe and Elementary Particles, Chonnam National University, Kwangju, Korea; 95 Korea University, Seoul, Korea; 96 University of Seoul, Seoul, Korea; 97 Sungkyunkwan University, Suwon, Korea; 98 Vilnius University, Vilnius, Lithuania; 99 Centro de Investigacion y de Estudios Avanzados del IPN, Mexico City, Mexico; 100 Universidad Iberoamericana, Mexico City, Mexico; 101 Benemerita Universidad Autonoma de Puebla, Puebla, Mexico; 102 Universidad Autónoma de San Luis Potosí, San Luis Potosí, Mexico; 103 University of Auckland, Auckland, New Zealand; 104 University of Canterbury, Christchurch, New Zealand; 105 National Centre for Physics, Quaid-I-Azam University, Islamabad, Pakistan; 106 National Centre for Nuclear Research, Swierk, Poland; 107 Institute of Experimental Physics, Faculty of Physics, University of Warsaw, Warsaw, Poland; 108 Laboratório de Instrumentação e Física Experimental de Partículas, Lisboa, Portugal; 109 Joint Institute for Nuclear Research, Dubna, Russia; 110 Petersburg Nuclear Physics Institute, Gatchina (St. Petersburg), Russia; 111 Institute for Nuclear Research, Moscow, Russia; 112 Institute for Theoretical and Experimental Physics, Moscow, Russia; 113 P.N. Lebedev Physical Institute, Moscow, Russia; 114 Skobeltsyn Institute of Nuclear Physics, Lomonosov Moscow State University, Moscow, Russia; 115 State Research Center of Russian Federation, Institute for High Energy Physics, Protvino, Russia; 116 University of Belgrade, Faculty of Physics and Vinca Institute of Nuclear Sciences, Belgrade, Serbia; 117 Centro de Investigaciones Energéticas Medioambientales y Tecnológicas (CIEMAT), Madrid, Spain; 118 Universidad Autónoma de Madrid, Madrid, Spain; 119 Universidad de Oviedo, Oviedo, Spain; 120 Instituto de Física de Cantabria (IFCA), CSIC-Universidad de Cantabria, Santander, Spain; 121 European Organization for Nuclear Research, CERN, Geneva, Switzerland; 122 Paul Scherrer Institut, Villigen, Switzerland; 123 Institute for Particle Physics, ETH Zurich, Zurich, Switzerland; 124 Universität Zürich, Zurich, Switzerland; 125 National Central University, Chung-Li, Taiwan; 126 National Taiwan University (NTU), Taipei, Taiwan; 127 Chulalongkorn University, Bangkok, Thailand; 128 Cukurova University, Adana, Turkey; 129 Physics Department, Middle East Technical University, Ankara, Turkey; 130 Bogazici University, Istanbul, Turkey; 131 Istanbul Technical University, Istanbul, Turkey; 132 Kharkov Institute of Physics and Technology, National Scientific Center, Kharkov, Ukraine; 133 University of Bristol, Bristol, United Kingdom; 134 Rutherford Appleton Laboratory, Didcot, United Kingdom; 135 Imperial College, London, United Kingdom; 136 Brunel University, Uxbridge, United Kingdom; 137 Baylor University, Waco, USA; 138 The University of Alabama, Tuscaloosa, USA; 139 Boston University, Boston, USA; 140 Brown University, Providence, USA; 141 University of California, Davis, Davis, USA; 142 University of California, Los Angeles, USA; 143 University of California, Riverside, Riverside, USA; 144 University of California, San Diego, La Jolla, USA; 145 University of California, Santa Barbara, Santa Barbara, USA; 146 California Institute of Technology, Pasadena, USA; 147 Carnegie Mellon University, Pittsburgh, USA; 148 University of Colorado at Boulder, Boulder, USA; 149 Cornell University, Ithaca, USA; 150 Fairfield University, Fairfield, USA; 151 Fermi National Accelerator Laboratory, Batavia, USA; 152 University of Florida, Gainesville, USA; 153 Florida International University, Miami, USA; 154 Florida State University, Tallahassee, USA; 155 Florida Institute of Technology, Melbourne, USA; 156 University of Illinois at Chicago (UIC), Chicago, USA; 157 The University of Iowa, Iowa City, USA; 158 Johns Hopkins University, Baltimore, USA; 159 The University of Kansas, Lawrence, USA; 160 Kansas State University, Manhattan, USA; 161 Lawrence Livermore National Laboratory, Livermore, USA; 162 University of Maryland, College Park, USA; 163 Massachusetts Institute of Technology, Cambridge, USA; 164 University of Minnesota, Minneapolis, USA; 165 University of Mississippi, Oxford, USA; 166 University of Nebraska-Lincoln, Lincoln, USA; 167 State University of New York at Buffalo, Buffalo, USA; 168 Northeastern University, Boston, USA; 169 Northwestern University, Evanston, USA; 170 University of Notre Dame, Notre Dame, USA; 171 The Ohio State University, Columbus, USA; 172 Princeton University, Princeton, USA; 173 University of Puerto Rico, Mayaguez, USA; 174 Purdue University, West Lafayette, USA; 175 Purdue University Calumet, Hammond, USA; 176 Rice University, Houston, USA; 177 University of Rochester, Rochester, USA; 178 The Rockefeller University, New York, USA; 179 Rutgers, The State University of New Jersey, Piscataway, USA; 180 University of Tennessee, Knoxville, USA; 181 Texas A&M University, College Station, USA; 182 Texas Tech University, Lubbock, USA; 183 Vanderbilt University, Nashville, USA; 184 University of Virginia, Charlottesville, USA; 185 Wayne State University, Detroit, USA; 186 University of Wisconsin, Madison, USA

## Abstract

Characteristics of multi-particle production in proton-proton collisions at $\sqrt{s}=7\ \mbox{TeV}$ are studied as a function of the charged-particle multiplicity, *N*_ch_. The produced particles are separated into two classes: those belonging to jets and those belonging to the underlying event. Charged particles are measured with pseudorapidity |*η*|<2.4 and transverse momentum *p*_T_>0.25 GeV/*c*. Jets are reconstructed from charged-particles only and required to have *p*_T_>5 GeV/*c*. The distributions of jet *p*_T_, average *p*_T_ of charged particles belonging to the underlying event or to jets, jet rates, and jet shapes are presented as functions of *N*_ch_ and compared to the predictions of the pythia and herwig event generators. Predictions without multi-parton interactions fail completely to describe the *N*_ch_-dependence observed in the data. For increasing *N*_ch_, pythia systematically predicts higher jet rates and harder *p*_T_ spectra than seen in the data, whereas herwig shows the opposite trends. At the highest multiplicity, the data–model agreement is worse for most observables, indicating the need for further tuning and/or new model ingredients.

## Introduction

Achieving a complete understanding of the details of multi-particle production in hadronic collisions remains an open problem in high-energy particle physics. In proton-proton (pp) collisions at the energies of the Large Hadron Collider (LHC), most of the inelastic particle production is described in a picture in which an event is a combination of hadronic jets, originating from hard parton-parton interactions with exchanged momenta above several GeV/*c*, and of an underlying event consisting of softer parton-parton interactions, and of proton remnants.

The production of high-transverse-momentum jets, defined as collimated bunches of hadrons, results from parton cascades generated by the scattered quarks and gluons, described by perturbative quantum chromodynamics (QCD), followed by non-perturbative hadronization described either via color fields (“strings”) stretching between final partons, or by the formation of colorless clusters of hadrons [1]. The underlying event (UE) is commonly defined as the set of all final-state particles that are not associated with the initial hard-parton scattering. This component is presumably dominated by perturbative (mini)jets with relatively small transverse momenta of a few GeV/*c*, produced in softer multi-parton interactions (MPI) [2–8], as well as by soft hadronic strings from the high-rapidity remnants. The description of the UE is more phenomenological than that of the jets arising from the primary hard-parton scatter, whose final hadron multiplicity can be in principle computed in QCD [1]. In this two-component approach, rare high-multiplicity events can be explained as due to a large number of MPI taking place in the pp collisions at small impact parameters. Different variants of such a physical picture are realized in state-of-the-art Monte Carlo (MC) event generators such as pythia [9, 10] and herwig [11, 12]. The properties of multi-particle production are very sensitive to the assumptions made about the combination of MPI and hard scatterings, the modeling of the multi-parton interactions (in particular the transverse structure of the proton) [3], and non-perturbative final-state effects such as color reconnections, hadronization mechanisms, and possible collective-flow phenomena, among others.

Experimental data on multi-particle production in pp collisions at LHC energies provide a clear indication that our understanding of the different components contributing to the total inelastic cross section is incomplete. This arises from difficulties in describing multiplicity distributions, and especially the high-multiplicity tails [13], or in reproducing a new structure of the azimuthal angular correlations at 7 TeV for high-multiplicity events, the so-called “ridge” [14]. Interesting disagreements between data and MC simulation were also recently reported in transverse sphericity analyses and for global event shapes [15–17]. Together with similar findings in nucleus-nucleus collisions, these disagreements point to the intriguing possibility of some mechanisms at high multiplicities which are not properly accounted for in event generator models. Therefore, although the standard mixture of (semi)hard and non-perturbative physics considered by pythia and herwig is often sufficient for reproducing the bulk properties of inelastic events, it fails to provide a more detailed description of the data and in particular of the properties of events binned in particle multiplicity.

The average transverse momentum of the charged particles produced in pp and $\mathrm{p}\bar{\mathrm{p}}$ collisions has been measured as a function of the event multiplicity at various center-of-mass energies [13, 18–22]. The work presented here is the first one that carries out the study also for the UE and jets separately and includes other observables (jet *p*_T_ spectra, rates and shapes) not analyzed before as a function of particle multiplicity with such a level of detail.

The paper is organized as follows. The general procedure of the analysis is described in Sect. 2, a short description of the Compact Muon Solenoid (CMS) detector is given in Sect. 3, and the event generator models used are presented in Sect. 4. Sections 5 to 7 describe trigger and event selection, track and jet reconstruction, the data correction procedure, and the systematic uncertainties. Results and discussions are presented in Sect. 8, and summarized in Sect. 9.

## Analysis strategy

The main goal of this analysis is to study the characteristic features and relative importance of different mechanisms of multi-particle production in pp collisions at a center-of-mass energy of $\sqrt{s}=7\ \mbox{TeV}$ in different charged-particle multiplicity bins, corresponding to different levels of hadronic activity resulting from larger or smaller transverse overlap of the colliding protons. Guided by the two-component physical picture described in the introduction, we separate the particle content of each inelastic event into two subsets. We identify the jet-induced contribution and treat the rest as the underlying event originating from unresolved perturbative sources such as semihard MPI and other softer mechanisms. Our approach to this problem uses the following procedure, applied at the stable (lifetime *cτ*>10 mm) particle-level:

- Similarly to the centrality classification of events in high-energy nuclear collisions [23], events are sorted according to their charged-particle multiplicity (Table 1). Hereafter, for simplicity, multiplicity should always be understood as charged-particle multiplicity.

**Table 1:** Charged-particle multiplicity bins, mean charged-particle multiplicity in bins, and corresponding number of events. The multiplicity *N*_ch_ is defined as the total number of stable charged-particles in the events, corrected for inefficiencies, with transverse momentum *p*_T_>0.25 GeV/*c* and pseudorapidity |*η*|<2.4

Multiplicity range	Mean multiplicity 〈*N*_ch_〉	Number of events
10<*N*_ch_≤30	18.9	2 795 688
30<*N*_ch_≤50	38.8	1 271 987
50<*N*_ch_≤80	61.4	627 731
80<*N*_ch_≤110	90.6	105 660
110<*N*_ch_≤140	120	11 599
- For each event, jets are built with charged particles only using the anti-*k*_T_ algorithm [24, 25] with a distance parameter 0.5, optimized as described below, and are required to have a *p*_T_>5 GeV/*c*. Charged particles falling within a jet cone are labeled as “intrajet particles”.
- After removing all intrajet particles from the event, the remaining charged particles are defined as belonging to the underlying event. Events without jets above *p*_T_=5 GeV/*c* are considered to consist of particles from the UE only.

In order to achieve a better separation of the contributions due to jets and underlying event, the resolution parameter of the anti-*k*_T_ algorithm is increased until the UE charged-particle *p*_T_-spectrum starts to saturate, indicating that the jet component has been effectively removed. This way of fixing the jet cone radius minimizes contamination of the underlying event by jet contributions or vice versa. A resolution parameter of value 0.5 is found to be optimal. Of course, it is not possible to completely avoid mixing between jets and underlying event. To clarify the picture and minimize the mixing of the two components, we measure not only the *p*_T_ spectrum of the charged particles inside jet cones, but also the spectrum of the leading (the highest-*p*_T_) charged particle in each cone.

## The CMS detector

A detailed description of the CMS detector can be found in Ref. [26]. A right-handed coordinate system with the origin at the nominal interaction point (IP) is used, with the *x* axis pointing to the center of the LHC ring, the *y* axis pointing up, and the *z* axis oriented along the anticlockwise-beam direction. The central feature of the CMS detector is a superconducting solenoid of 6 m internal diameter providing an axial magnetic field with a nominal strength of 3.8 T. Immersed in the magnetic field are the pixel tracker, the silicon-strip tracker, the lead tungstate electromagnetic calorimeter, the brass/scintillator hadron calorimeter, and the muon detection system. In addition to the barrel and endcap calorimeters, the steel/quartz-fibre forward calorimeter covers the pseudorapidity region 2.9<|*η*|<5.2, where *η*=−log[tan(*θ*/2)], and *θ* is the polar angle measured at the center of the CMS detector with respect to the *z* axis. The tracking detector consists of 1440 silicon-pixel and 15 148 silicon-strip detector modules. The barrel part consists of 3 (10) layers of pixel (strip) modules around the IP at distances ranging from 4.4 cm to 1.1 m. Five out of the ten strip layers are double-sided and provide additional *z* coordinate measurements. The two endcaps consist of 2 (12) disks of pixel (strip) modules that extend the pseudorapidity acceptance to |*η*|=2.5. The tracker provides an impact parameter resolution of about 100 μm and a *p*_T_ resolution of about 0.7 % for 1 GeV/*c* charged particles at normal incidence. Two of the CMS subdetectors acting as LHC beam monitors, the Beam Scintillation Counters (BSC) and the Beam Pick-up Timing for the eXperiments (BPTX) devices, are used to trigger the detector readout. The BSC are located along the beam line on each side of the IP at a distance of 10.86 m and cover the range 3.23<|*η*|<4.65. The two BPTX devices, which are located inside the beam pipe and ±175 m from the IP, are designed to provide precise information on the structure and timing of the LHC beams with a time resolution of 0.2 ns.

## Event generator models

The best available general-purpose event generators and their tunes are used for comparison with the data. They are the pythia 6 (version 6.424 [9], tune Z2*), pythia 8 (version 8.145 [10], tune 4C [27]), and herwig++ 2.5 (tune UE-EE-3M) [12] event generators. These event generators and tunes differ in the treatment of initial and final state radiation, hadronization, and in the choice of underlying event parameters, color reconnections, and cutoff values for the MPI mechanism. Values of these parameters were chosen to provide a reasonable description of existing LHC pp differential data measured in minimum-bias and hard QCD processes. Initial and final state radiation is essential for the correct description of jet production and of the UE [28]. For the MPI modeling, pythia incorporates interleaved evolution between the different scatterings [27, 29], whereas herwig concentrates more hard scatterings at the center of the pp collision while allowing for more (disconnected) soft-parton scatterings at the periphery. A detailed review of the implementation of all these mechanisms in modern MC event generators is given in [30]. The most recent pythia 6 Z2* tune is derived from the Z1 tune [31], which uses the CTEQ5L parton distribution set, whereas Z2* adopts CTEQ6L [32]. The Z2* tune is the result of retuning the pythia parameters PARP(82) and PARP(90) by means of the automated Professor tool [33], yielding PARP(82)=1.921 and PARP(90)=0.227 GeV/*c*. The results of this study are also compared to predictions obtained with pythia 8, tune 4C, with multi-parton interactions switched off. Hadronization in pythia is based on the Lund string model [2] while that in herwig is based on the cluster fragmentation picture in which perturbative evolution forms preconfined clusters that subsequently decay into final hadrons. The version of herwig++ 2.5 UE-EE-3M used in this paper includes important final-state effects due to color reconnections and is based on the MRST2008 parton distribution set [34].

## Event selection and reconstruction

The present analysis uses the low-pileup data recorded during the first period of 2010 data taking, corresponding to an integrated luminosity of (3.18±0.14) pb^−1^. The data are collected using a minimum-bias trigger requiring a signal from both BPTX detectors coincident with a signal from both BSC detectors.

For this analysis, the position of the reconstructed primary vertex is constrained to be within ±10 cm with respect to the nominal IP along the beam direction and within ±2 cm in the transverse direction, thereby substantially rejecting non-collision events [35]. The fraction of background events after these selections is found to be negligible (<0.1 %).

The fraction of events in the data sample with pileup (two or more pp collisions per bunch crossing) varies in the range (0.4–7.8) % depending on the instantaneous luminosity per bunch. This small fraction of pileup events is kept, but the analysis is only carried out for the tracks connected with the primary (highest multiplicity) vertex. The fraction of events where two event vertices are reconstructed as one, or where two event vertices share associated tracks, ranges between (0.04–0.2) %.

### Track reconstruction and selection

The track reconstruction procedure uses information from both pixel and strip detectors and is based on an iterative combinatorial track finder [36]. Tracks are selected for analysis if they have transverse momenta *p*_T_>0.25 GeV/*c* and pseudorapidities lying within the tracker acceptance |*η*|<2.4. Such *p*_T_ cut provides robust measurements, keeping the event selection minimally biased by hard processes. In addition, tracks must be associated with the event vertex with the highest multiplicity in the bunch crossing. The requirement removes tracks coming from secondary interactions with detector materials, decays of long-lived neutral hadrons, and pileup. Residual contamination from such tracks is at the level of 0.2 %.

### Charged-particle jet reconstruction

This analysis is based on jets that are reconstructed using tracks only, in order to avoid the reconstructed jet energy uncertainty due to mismeasurements of low-*p*_T_ neutral particles. Jets are reconstructed by clustering the tracks with the collinear- and infrared-safe anti-*k*_T_ algorithm with a distance parameter of 0.5, that results in cone-shaped jets. Jets are retained if their axes lie within the fiducial region |*η* ^jet axis^|<1.9, so that for a jet with an effective radius of 0.5 all jet constituent tracks fall within the tracker acceptance (|*η*|<2.4).

## Data correction

### Event selection efficiency

In the MC simulations, events are selected at the stable-particle level (lifetime *cτ*>10 mm) if at least one charged particle is produced on each side of the interaction point within 3.32<|*η*|<4.65, mimicking the BSC trigger requirement, and, in addition, if at least five charged particles with *p*_T_>0.25 GeV/*c* and |*η*|<2.4 are present, which ensures a high vertex finding efficiency in the offline selection of data.

The trigger efficiency is measured using data collected with a zero-bias trigger, constructed from a coincidence of the BPTX counters, which effectively requires only the presence of colliding beams at the interaction point. The offline selection efficiency is determined from MC simulations. The combined trigger and offline selection efficiency is obtained as a function of the number of reconstructed tracks and is very high: above 87 % for events with more than 10 reconstructed tracks and close to 100 % for events with more than 30 reconstructed tracks. Results are corrected by applying a weight inversely proportional to the efficiency for each observed event.

### Corrections related to the track reconstruction

The track-based quantities (*N*_ch_, average *p*_T_ of tracks, jet *p*_T_ density in ring zones) are corrected in a two-stage correction procedure. First, each observed track is given a weight to account for track reconstruction inefficiencies and misreconstructed (fake) track rates, as obtained from the detector simulation. The weights are based on two-dimensional matrices *ϵ*(*η*,*p*_T_) and *f*(*η*,*p*_T_), for reconstruction efficiency and fake track rates, respectively, computed in bins in *η*, *p*_T_, and is given by

1 $$ N^\text{true}_\text{ch}(\eta, p_{\mathrm{T}})=N^{\text {reco}}_\text{ch}( \eta, p_{\mathrm{T}})\frac{{1-f(\eta, p_{\mathrm{T}})}}{\epsilon(\eta, p_{\mathrm{T}})}. $$

The corrections for reconstruction efficiencies and fake rates depend on track multiplicity. Therefore, four different sets of matrices *ϵ*(*η*,*p*_T_) and *f*(*η*,*p*_T_) for different track multiplicity classes are used, the first three track multiplicity classes corresponding to the first three charged-particle multiplicity bins of Table 1 and the fourth one corresponding to the fourth and fifth charged-particle multiplicity bins. The average track reconstruction efficiency and fake rate vary between 79–80 % and 3–4 %, respectively, depending on the multiplicity bin considered.

Table 1 shows the corrected charged-particle multiplicity classes used in this analysis and the number of events and mean multiplicities in each multiplicity bin after applying all event selection criteria.

**Fig. 1:**
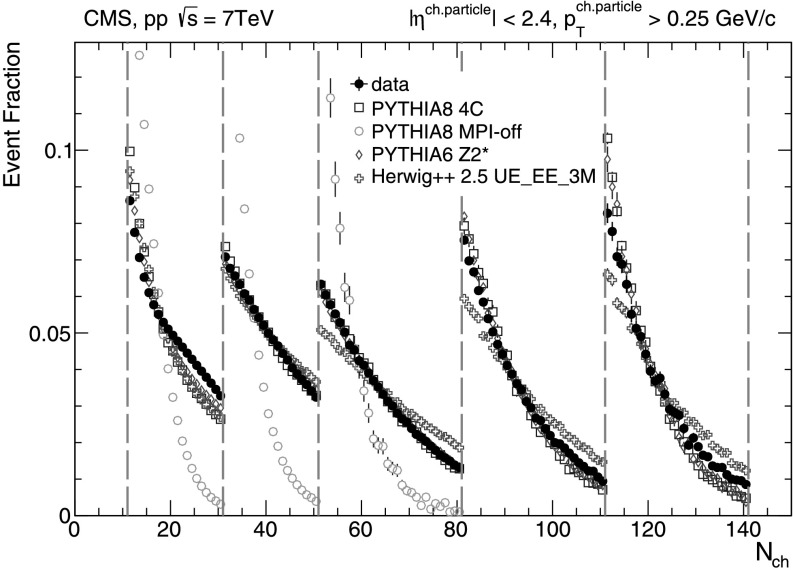
Charged-particle multiplicity distributions, corrected for tracking efficiency and fake rate, for the five multiplicity bins defined in this analysis compared to four different MC predictions. The normalization is done for each multiplicity bin separately. pythia 8 with MPI switched off completely fails to produce events at large multiplicity and therefore no points are shown in the two highest multiplicity domains

Figure 1 shows multiplicity distributions that have been corrected for tracking efficiency and fake rate. The simulations fail to describe all the measured *N*_ch_ distributions, as discussed in Ref. [13]. As we are considering event properties as a function of multiplicity, such a data–MC disagreement might introduce a bias due to the different *N*_ch_ distribution within the wide multiplicity intervals. Reweighting the multiplicity distributions in MC simulations to bring them in agreement with the ones observed in data results in less than 1–2 % corrections for all results. In the following, corrected results are compared to the predictions obtained from the unweighted MC models.

All the measured quantities hereafter are further corrected to stable-particle level using a bin-by-bin factor obtained from Monte Carlo simulations. This correction factor accounts for event migration between adjacent multiplicity bins, for differences in the tracking performance in the dense environment inside jets, and for mixing between charged particles belonging to charged-particle jets and the UE due to jets that are misidentified at the detector level. The magnitude of this correction factor is typically less than 1 %, except for the jet *p*_T_ density in the core of the jet where it reaches up to 8 %.

### Correction of the track-jet *p*_T_ distributions

Track-jet distributions have to be corrected for inefficiencies in reconstruction, for misidentified jets, and for bin migrations due to the finite energy resolution. On average, a reconstructed track-jet has 95 % of the energy of the original charged-particle jet. The energy resolution of such jets is about 13 %. The reconstructed jet spectrum is related to the “true” jet spectrum as follows:

2 $$ M\bigl(p_{\mathrm{T}}^\text{measured}\bigr) = \int C \bigl(p_{\mathrm{T}}^\text {measured},p_{\mathrm{T}}^\text {true}\bigr)T\bigl(p_{\mathrm{T}}^\text{true}\bigr)\, \mathrm{d}p_{\mathrm {T}}^\text{true}, $$

where $M(p_{\mathrm{T}}^{\text{measured}})$ and $T(p_{\mathrm {T}}^{\text{true}})$ are the measured and the true *p*_T_ spectra, respectively, and $C(p_{\mathrm{T}}^{\text{measured}},p_{\mathrm{T}}^{\text{true}})$ is a response function obtained from the MC simulation. The problem of inverting the response relation of Eq. (2) is well known and has been extensively studied in literature. In our analysis, an iterative unfolding technique [37] is applied. Since the detector response changes with multiplicity, individual response matrices are used for each multiplicity bin.

## Systematic uncertainties

The following sources of systematic uncertainties are considered:

### Association of tracks with the primary vertex (track selection)

Tracks that are coming from a non-primary interaction result in an incorrect multiplicity classification of the event and bias the event properties at a given multiplicity. These tracks originate from secondary interactions with detector material, decays of long-lived neutral hadrons, and pileup. Moreover, these tracks can bias the *p*_T_ spectrum of primary tracks. As it is not possible to completely avoid contamination by such tracks, the stability of the results has been estimated by tightening and loosening the association criteria. Removing contamination inevitably leads to rejection of some valid primary tracks, so for each set of the association criteria a special efficiency and fake-rate correction must be used.

### Tracking performance

A correct description of the tracking performance in the MC simulation of the detector is essential. A conservative estimate of the uncertainty of this efficiency of 2.3 % is taken from Ref. [38].

### Model dependence of the correction procedures

Different MC models can give slightly different detector and reconstruction responses. Two models, pythia 6 tune Z2* and pythia 8 tune 4C, are used to compute tracking and jet performance and correction factors. herwig++ 2.5 was found to deviate too much from the data and was not used for the estimate of the systematic uncertainty. Corrections based on the pythia 6 tune Z2* model, which provides better agreement with data, are used to get the central values of different physics quantities. The differences between these two methods are assigned as a systematic uncertainty.

### Unfolding the jet *p*_T_ spectrum

The unfolding procedure used to correct for bin migrations in the jet *p*_T_ spectra is based on an iterative unfolding technique [37] for which we find that 4–5 iterations are optimal. By varying the number of iterations (±1 with respect to the optimal value) and the reconstructed-to-generated jet matching parameter (0.15<Δ*R*<0.25) we obtain a systematic uncertainty of (0.5–2.0) %. This leads to a systematic uncertainty <0.2 % in the average *p*_T_ of the jet spectrum, and <2 % for charged-particle jet rates.

Although this analysis uses a low-pileup data sample, rare high-multiplicity events might occur due to overlapping pp collisions in the same bunch crossing. The effect of pileup is estimated by comparing results at different instantaneous luminosities. The dataset is divided into subsets according to the instantaneous luminosity and the differences found between these subsets are of the order of the statistical uncertainties of the sample. In addition, it was checked that the instantaneous luminosity for events with small and large *N*_ch_ does not differ, confirming that the large-multiplicity bins are not biased by a possibly increased contribution from pileup events. Therefore, we conclude that high-multiplicity events are not affected by pileup.

Tables 2 and 3 summarize the systematic and statistical uncertainties of the measured quantities. The total uncertainties are the sum in quadrature of the individual systematic and statistical uncertainties. The total error of jet *p*_T_ density as a function of jet radius rises with *R* and *N*_ch_. The total uncertainties in the jet *p*_T_ spectra are of the order of 4–8 % for jet *p*_T_ up to about 25 GeV/*c*. For jets with *p*_T_>25 GeV/*c* the statistical uncertainties dominate.

**Table 2:** Summary of systematic and statistical uncertainties for various averaged inclusive and UE-related quantities. The variables $\langle p_{\mathrm{T}}^{\text{ch. particle}} \rangle$, $\langle p_{\mathrm{T}}^{\mathrm{UE}} \rangle$, 〈*PT* ^ij^〉, $\langle p_{\mathrm{T}}^{\mathrm{ijl}} \rangle$ are defined in Sect. 8.1, *ρ*(*R*) is defined in Sect. 8.2.3

	$\langle p_{\mathrm{T}}^{\text{ch. particle}} \rangle$	$\langle p_{\mathrm{T}}^{\mathrm{UE}} \rangle$	$\langle p_{\mathrm{T}}^{\mathrm{ij}} \rangle$	$\langle p_{\mathrm {T}}^{\mathrm{ijl}} \rangle$	*ρ*(*R*)
Track selection	<0.2 %	<0.2 %	<0.2 %	<0.4 %	<1 %
Tracking performance	<0.3 %	<0.3 %	<0.4 %	<0.4 %	<4 %
Model dependence	<0.5 %	<0.4 %	<0.5 %	<0.5 %	<5 %
Statistical	<0.1 %	<0.1 %	<0.2 %	<0.4 %	2–8 %
Total	0.5–0.7 %	0.5–0.6 %	0.5–0.7 %	<0.9 %	4–9 %

**Table 3:** Summary of systematic and statistical uncertainties for various charged-jet related quantities

	ch. jet *p*_T_ spectrum	ch. jet rate (*p*_T_>5 GeV/*c*)	ch. jet rate (*p*_T_>30 GeV/*c*)	$\langle p_{\mathrm{T}}^{\text{ch. jet}}\rangle$
Track selection	<1 %	<2 %	<4 %	<0.1 %
Tracking performance	<3 %	2 %	<5 %	<0.5 %
Model dependence	<3 %	2 %	<6 %	<0.4 %
Unfolding	3 %	<2 %	<3 %	<0.2 %
Statistical	1–8 % $(p_{\mathrm{T}}^{\text{ch. jet}}<25~\text{GeV/}c)$ 10–40 % $(p_{\mathrm{T}}^{\text{ch. jet}}>25~\text{GeV/}c)$	<1 %	<9 %	<0.4 %
Total	4–10 % $(p_{\mathrm{T}}^{\text{ch. jet}}<25~\text{GeV/}c)$ 10–40 % $(p_{\mathrm{T}}^{\text{ch. jet}}>25~\text{GeV/}c)$	<5 %	<12 %	0.8 %

## Results

### General properties of charged particles from jets and from the UE

We start with discussing the general jet and UE properties in the five *N*_ch_ bins defined. Tables 4, 5 list the average transverse momentum for the various types of charged particles measured, as well as the predictions from pythia 8 tune 4C, pythia 8 MPI-off, pythia 6 tune Z2*, and herwig++ 2.5. For each multiplicity bin, we show the fully corrected results for the mean transverse momenta of all charged particles $\langle p_{\mathrm {T}}^{\text{ch. particle}} \rangle$, UE charged particles $\langle p_{\mathrm{T}}^{\mathrm{UE}} \rangle$, intrajet charged particles $\langle p_{\mathrm{T}}^{\mathrm{ij}} \rangle$, intrajet leading charged particles $\langle p_{\mathrm{T}} ^{\mathrm{ijl}} \rangle$, the mean transverse momentum of charged-particle jets $\langle p_{\mathrm{T}}^{\text{ch. jet}} \rangle$, and the average number of jets per event $\langle\frac{\#\text{jets}}{\text{event}} \rangle$.

**Table 4:** Average transverse momenta for different types of charged particles (inclusive, underlying event, intrajet, leading intrajet). The quantities are compared with the MC predictions. Uncertainties smaller than the last significant digit are omitted

	$\langle p_{\mathrm{T}}^{\text{ch. particle}} \rangle,~\text{GeV/}c$	$\langle p_{\mathrm{T}}^{\mathrm{UE}} \rangle,~\text{GeV/}c$	$\langle p_{\mathrm{T}}^{\mathrm{ij}} \rangle,~\text{GeV/}c$	$\langle p_{\mathrm{T}}^{\mathrm{ijl}} \rangle,~\text{GeV/}c$
10<*N*_ch_≤30				
Data	0.68±0.01	0.65±0.01	1.90±0.02	3.65±0.05
pythia 8 4C	0.67	0.64	1.83	3.48±0.01
pythia 8 MPI-off	0.72	0.66	1.93	3.73
pythia 6 Z2*	0.67	0.65	1.86	3.59
herwig++ 2.5	0.68	0.65	1.81	3.41
30<*N*_ch_≤50				
Data	0.75±0.01	0.71±0.01	1.64±0.02	3.37±0.04
pythia 8 4C	0.77	0.72	1.62	3.25±0.01
pythia 8 MPI-off	1.06	0.75	1.99	4.28±0.02
pythia 6 Z2*	0.74	0.70	1.62	3.33
herwig++ 2.5	0.72	0.68	1.62	3.26
50<*N*_ch_≤80				
Data	0.80±0.01	0.74 ± 0.01	1.45±0.01	3.15±0.03
pythia 8 4C	0.84	0.76	1.49	3.10
pythia 8 MPI-off	1.47	0.80	2.22	5.17±0.09
pythia 6 Z2*	0.80	0.74	1.44	3.10
herwig++ 2.5	0.74	0.68	1.43	3.08
80<*N*_ch_≤110				
Data	0.85±0.01	0.76±0.01	1.32±0.01	2.96±0.03
pythia 8 4C	0.90	0.78	1.41	3.04±0.01
pythia 6 Z2*	0.85	0.76	1.33	2.97
herwig++ 2.5	0.74	0.66	1.28	2.94
110<*N*_ch_≤140				
Data	0.88±0.01	0.77±0.01	1.24±0.01	2.86±0.03
pythia 8 4C	0.95	0.79	1.36	3.05
pythia 6 Z2*	0.90	0.77	1.29	3.05±0.01
herwig++ 2.5	0.70	0.62	1.16	2.82±0.01

**Table 5:** Average transverse momentum of charged-particle jets and charged-particle jet rates for two thresholds, *p*_T_>5 GeV/*c* and *p*_T_> 30 GeV/*c*. The quantities are compared with the MC predictions. Uncertainties smaller than the last significant digit are omitted

	$\langle p_{\mathrm{T}}^{\text{ch. jet}}\rangle,~\text{GeV/}c$	$\langle\frac{\#\text{ch. jets}}{\text{event}} \rangle$ ($p_{\mathrm{T}} ^{\text{ch. jet}}>5~\text{GeV/}c$)	$\langle\frac{\#\text{ch. jets}}{\text{event}} \rangle$ ($p_{\mathrm{T}} ^{\text{ch. jet}}>30~\text{GeV/}c$)
10<*N*_ch_≤30			
Data	6.85±0.06	0.054±0.004	(3.2±0.5)10^−5^
pythia 8 4C	7.08±0.01	0.075	(3.9±0.6)10^−5^
pythia 8 MPI-off	7.96±0.01	0.152	(2.03±0.02)10^−4^
pythia 6 Z2*	7.01±0.01	0.067	(2.7±0.3)10^−5^
herwig++ 2.5	6.92±0.01	0.095	(3.8±0.5)10^−5^
30<*N*_ch_≤50			
Data	7.04 ± 0.09	0.287±0.014	(3.4±0.4)10^−4^
pythia 8 4C	7.26±0.01	0.386	(4.4±0.5)10^−4^
pythia 8 MPI-off	10.8	1.38 ± 0.02	(2.9±0.1)10^−2^
pythia 6 Z2*	7.20±0.01	0.304	(3.5±0.2)10^−4^
herwig++ 2.5	7.02±0.01	0.375	(3.1±0.3)10^−4^
50<*N*_ch_≤80			
Data	7.18±0.09	0.84±0.03	(1.5±0.1)10^−3^
pythia 8 4C	7.41±0.01	1.09	(1.8±0.1)10^−3^
pythia 8 MPI-off	16.3±0.4	3.1±0.3	(3.7±0.1)10^−1^
pythia 6 Z2*	7.30±0.01	0.87	(1.4±0.1)10^−3^
herwig++ 2.5	7.10±0.01	0.88	(5.9±0.5)10^−4^
80<*N*_ch_≤110			
Data	7.46±0.11	2.13±0.09	(4.3±0.4)10^−3^
pythia 8 4C	7.77±0.02	2.54	(7.1±0.6)10^−3^
pythia 6 Z2*	7.64±0.01	2.12	(5.7±0.2)10^−3^
herwig++ 2.5	7.25±0.01	1.66	(1.2±0.1)10^−3^
110<*N*_ch_≤140			
Data	7.81±0.10	3.68±0.15	(1.0±0.1)10^−2^
pythia 8 4C	8.31±0.03	4.46	(2.5±0.1)10^−2^
pythia 6 Z2*	8.15±0.02	3.95	(2.1±0.1)10^−2^
herwig++ 2.5	7.37 ± 0.01	2.41	(1.9±0.2)10^−3^

**Fig. 2:**
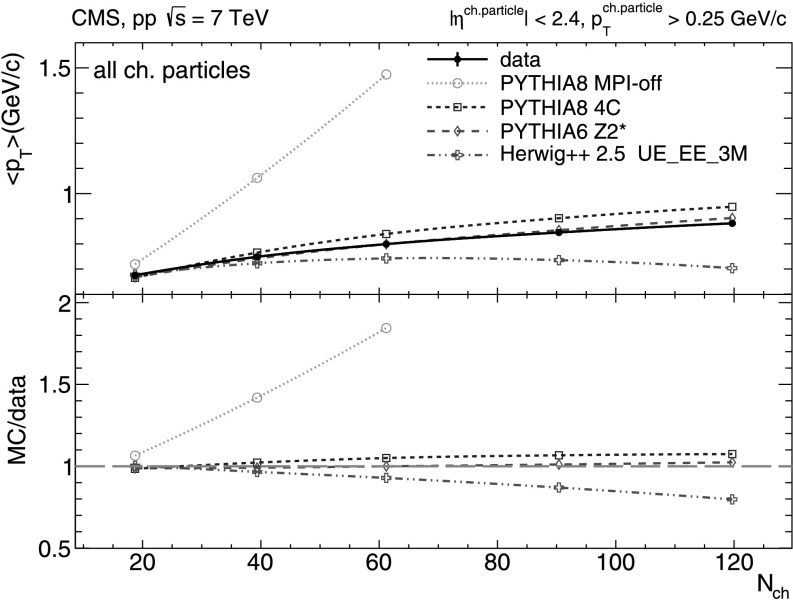
Mean transverse momentum of inclusive charged-particles with *p*_T_>0.25 GeV/*c* versus charged-particle multiplicity (*N*_ch_ within |*η*|< 2.4) measured in the data (*solid line* and *marker*) compared to various MC predictions (*non-solid curves* and *markers*). Systematic uncertainties are indicated by error bars which are, most of the time, smaller than the marker size

**Fig. 3:**
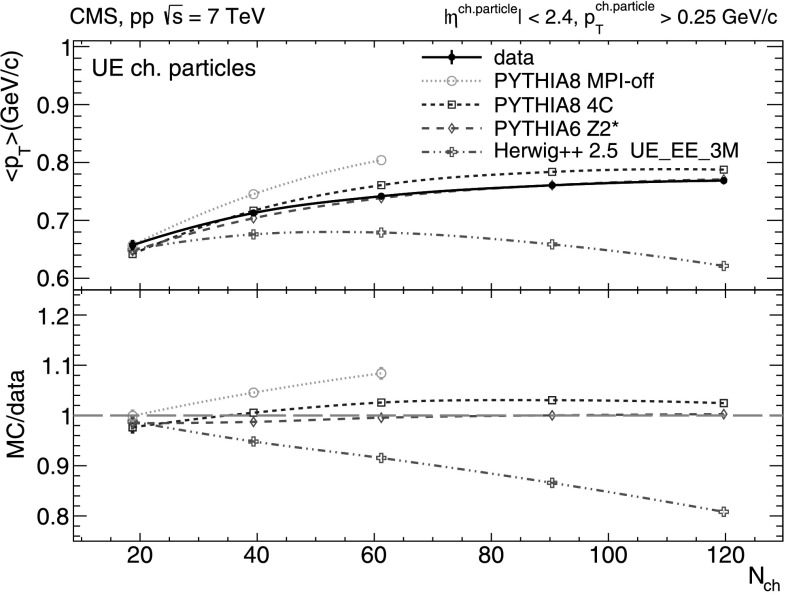
Mean transverse momentum of UE charged-particles with *p*_T_>0.25 GeV/*c* versus charged-particle multiplicity (*N*_ch_ within |*η*|< 2.4) measured in the data (*solid line* and *marker*) compared to various MC predictions (*non-solid curves* and *markers*). Systematic uncertainties are indicated by *error bars* which are, most of the time, smaller than the marker size

**Fig. 4:**
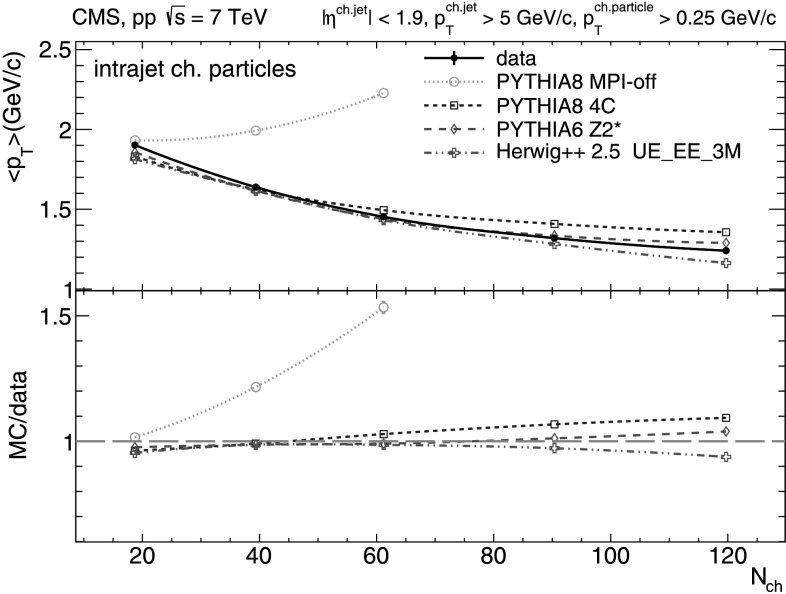
Mean transverse momentum of intrajet charged-particles with *p*_T_>0.25 GeV/*c* versus charged-particle multiplicity (*N*_ch_ within |*η*|<2.4) measured in the data (*solid line* and *marker*) compared to various MC predictions (*non-solid curves* and *markers*). Systematic uncertainties are indicated by *error bars* which are, most of the time, smaller than the marker size

The mean transverse momenta of all charged particles, UE charged-particles, and intrajet charged-particles, are plotted as a function of *N*_ch_ in Figs. 2–4. From Figs. 2 and 3, we see that mean transverse momentum of inclusive and UE charged-particles increases with *N*_ch_. Such a behavior is expected as the higher multiplicity events have an increased fraction of (semi)hard scatterings contributing to final hadron production. The (logarithmic-like) *N*_ch_-dependence of the average transverse momentum of inclusive and UE charged-particles is well described by both pythia 6 tune Z2* and pythia 8 tune 4C (especially by the former), and is less well described by herwig++ 2.5, which does not predict a monotonically rising dependence but a “turn down” beyond *N*_ch_≈ 60. On the other hand, pythia 8 without MPI fails to describe the data altogether, predicting much harder charged-particle spectra for increasing final multiplicity. This follows from the fact that pythia 8 without MPI can only produce high-multiplicity events through very hard jets with large intrajet multiplicity, instead of producing a larger number of semi-hard jets in the event.

**Fig. 5:**
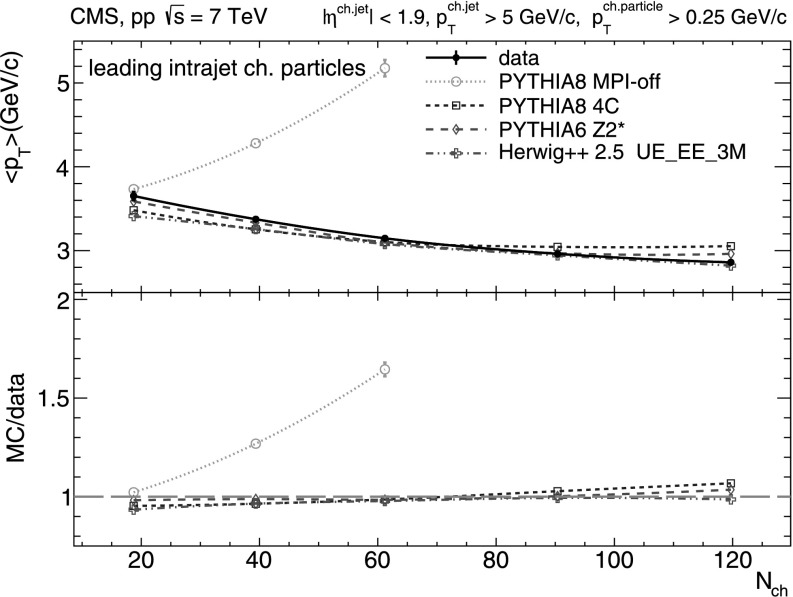
Mean transverse momentum of leading intrajet charged-particles with *p*_T_>0.25 GeV/*c* versus charged-particle multiplicity (*N*_ch_ within |*η*|<2.4) measured in the data (*solid line* and *marker*) compared to various MC predictions (*non-solid curves* and *markers*). Systematic uncertainties are indicated by *error bars* which are, most of the time, smaller than the marker size

From Figs. 4–5 it is clear that the *N*_ch_-dependence of the average *p*_T_ of intrajet constituents and leading charged-particle of the jets shows the opposite behavior compared to that from the global and underlying events (Figs. 2–3) and decreases logarithmically with increasing multiplicities. Events with increasing multiplicities are naturally “biased” towards final-states resulting mostly from (mini)jets which fragment into a (increasingly) large number of hadrons. Since the produced hadrons share the energy of the parent parton, a larger amount of them results in overall softer intrajet- and leading-hadron *p*_T_ spectra. Part of the decrease of the intrajet mean *p*_T_ with multiplicity could be also due to extra soft UE contribution falling within the jet cones, which increases from about 5 % for *N*_ch_≈20, to about 20 % for *N*_ch_≈120, according to pythia 6 tune Z2*. In terms of data-MC comparisons, we see that pythia 6 tune Z2* and herwig++ 2.5 describe relatively well the *N*_ch_-dependence of the intrajet and leading-particle average *p*_T_, whereas pythia 8 tune 4C produces harder mean charged-particle spectra at high multiplicities. The pythia 8 predictions without MPI increase dramatically with *N*_ch_, and fail to describe the data. This can be explained by the fact that pythia MPI-off enriches the increasing multiplicity range with events with hard partons only, whereas the other MC models include additional semi-hard parton interactions that soften the final hadron *p*_T_ spectra.

### Charged-particle jet properties

In the previous section, the jet substructure was investigated via the averaged properties of intrajet and leading particles. Now we turn to the description of the multiplicity-dependent properties of the jets themselves. In general, properties of inclusive jet production, when integrated over all multiplicities, are dominated by events with moderately low multiplicities, and are described quite well by QCD MC models [17, 39–41]. Here, we concentrate on the *N*_ch_-dependence of a subset of jet properties, such as the number of jets per event, the mean transverse momenta of jets, differential jet *p*_T_ spectra, and jet widths.

Our study is complementary to others based on global event shapes, e.g. from the ALICE experiment [15], which observed an increasing event transverse sphericity as a function of multiplicity in contradiction with the MC predictions. However, the corresponding multiplicities are much lower in the ALICE study than in this analysis because of their smaller rapidity coverage (|*η*|<0.8). Similar observations have been also recently seen by ATLAS [16], even though earlier CMS and ATLAS results show no serious disagreement with MC event generators [17, 40] as the events were not sorted according to their multiplicity. We show here that the higher sphericity of high-multiplicity events, relative to the pythia predictions, is due to an apparent reduction and softening of the jet yields at high-*N*_ch_.

#### Charged-particle jet production rates

**Fig. 6:**
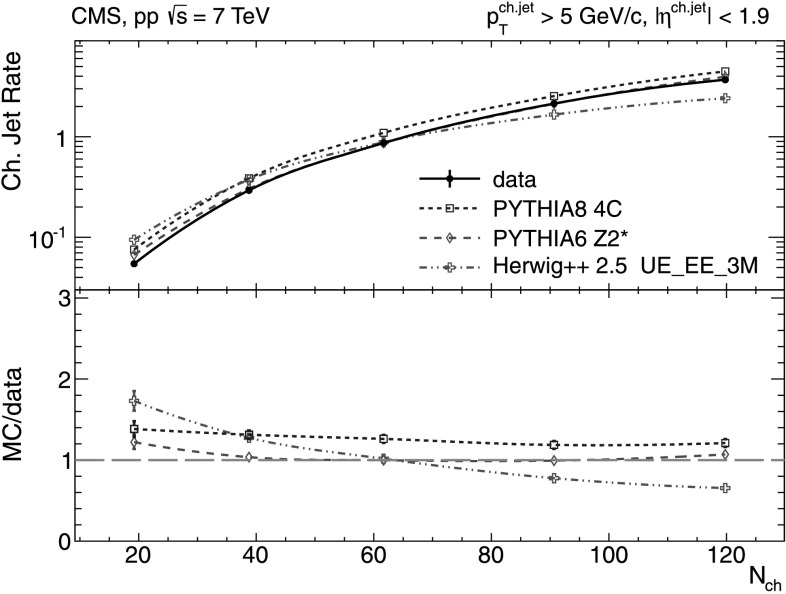
Number of charged-particle jets per event for $p_{\mathrm {T}}^{\text{ch. jet}}>5~\text{GeV/}c$ and jet axes lying within |*η*|<1.9 versus charged-particle multiplicity (*N*_ch_ within |*η*|<2.4) measured in the data (*solid line* and *marker*) compared to various MC predictions (*non-solid curves* and *markers*). Systematic uncertainties are indicated by *error bars* which are, most of the time, smaller than the marker size

**Fig. 7:**
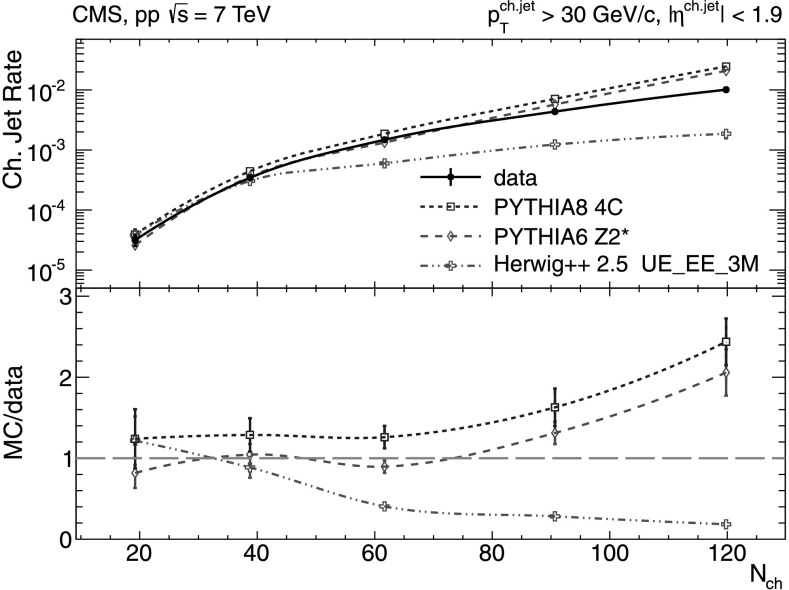
Number of charged-particle jets per event for $p_{\mathrm {T}}^{\text{ch. jet}}> 30~\text{GeV/}c$ and jet axes lying within |*η*|<1.9 versus charged-particle multiplicity (*N*_ch_ within |*η*|<2.4) measured in the data (*solid line* and *marker*) compared to various MC predictions (*non-solid curves* and *markers*). *Error bars* denote the total uncertainties

The *N*_ch_-dependence of the number of jets per event, with jet transverse momentum $p_{\mathrm{T}}^{\text{ch. jet}}>5~\text{GeV/}c$ and $p_{\mathrm {T}}^{\text{ch. jet}}>30~\text{GeV/}c$, is shown in Figs. 6 and 7, respectively.

For the small cutoff of 5 GeV/*c* the data show an increase from an average of 0.05 jets/event to about 4 jets/event going from the lowest to the highest charged-particle multiplicities. Such results, which confirm the importance of multiple (mini)jet production to explain the high-*N*_ch_ events, are very well described by pythia 6 tune Z2*, while predictions of pythia 8 tune 4C overestimate the rates at all *N*_ch_ and herwig++ 2.5 underestimates them for increasing *N*_ch_. For the higher 30 GeV/*c* cutoff, a large disagreement with the data is found in the higher-multiplicity bins (Fig. 7), where both versions of pythia predict a factor of two more jets per event than seen in the data. On the contrary, herwig++ 2.5 predicts a factor of 5 fewer jets per event than experimentally measured. The prediction of pythia 8 without MPI contributions is completely off-scale by factors of 3.5–6 above the data and is not shown in the plots.

**Fig. 8:**
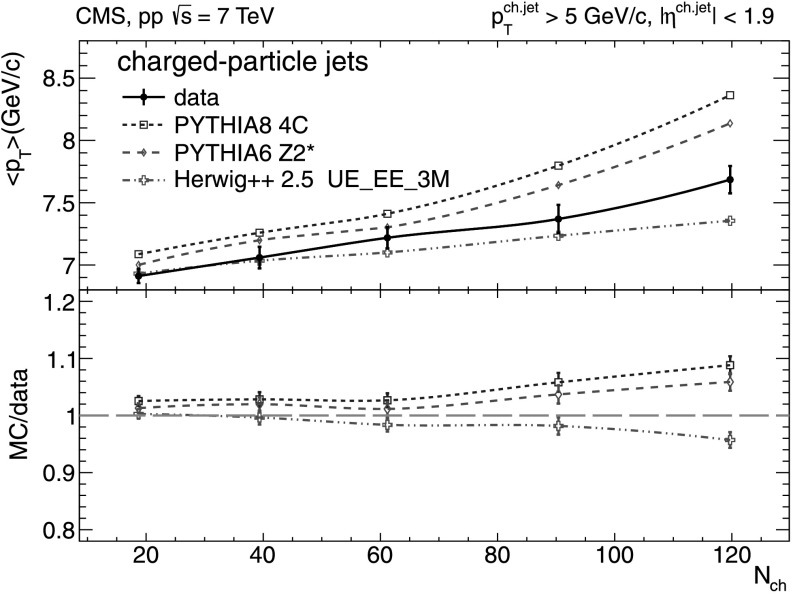
Mean transverse momentum of charged-particle jets with $p_{\mathrm{T}} ^{\text{ch. jet}}>5~\text{GeV/}c$ and jet axes within |*η*|<1.9) versus charged-particle multiplicity (*N*_ch_ within |*η*|<2.4) measured in the data (*solid line* and *marker*) compared to various MC predictions (*non-solid curves* and *markers*). *Error bars* denote the total uncertainties

The analysis of the *N*_ch_-dependence of the mean transverse momentum of charged-particle jets $\langle p_{\mathrm{T}}^{\text{ch. jet}} \rangle$ is shown in Fig. 8. The average $\langle p_{\mathrm{T}}^{\text{ch. jet}} \rangle$ rises slowly with *N*_ch_ from about 7.0 to 7.7 GeV/*c*, indicating a rising contribution from harder scatterings for increasingly “central” pp events. The predictions of pythia 8 tune 4C, pythia 6 tune Z2*, and herwig++ 2.5 are in good agreement with the data at low and intermediate multiplicities. However, the pythia models display an increasingly higher value of $\langle p_{\mathrm{T}}^{\text{ch. jet}} \rangle$, i.e. a harder jet contribution, up to 8.4 GeV/*c* in the highest-multiplicity events.

#### Charged-particle jet spectra

**Fig. 9:**
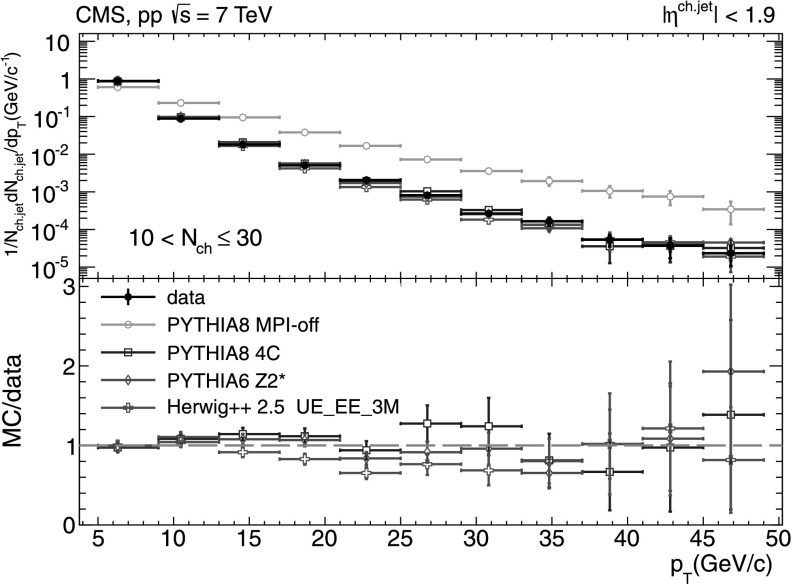
Inclusive charged-particle jet *p*_T_ spectrum for events with 10<*N*_ch_(|*η*|<2.4)≤30 measured in the data (*solid dots*) compared to various MC predictions (*empty markers*). *Error bars* denote the total uncertainties

**Fig. 10:**
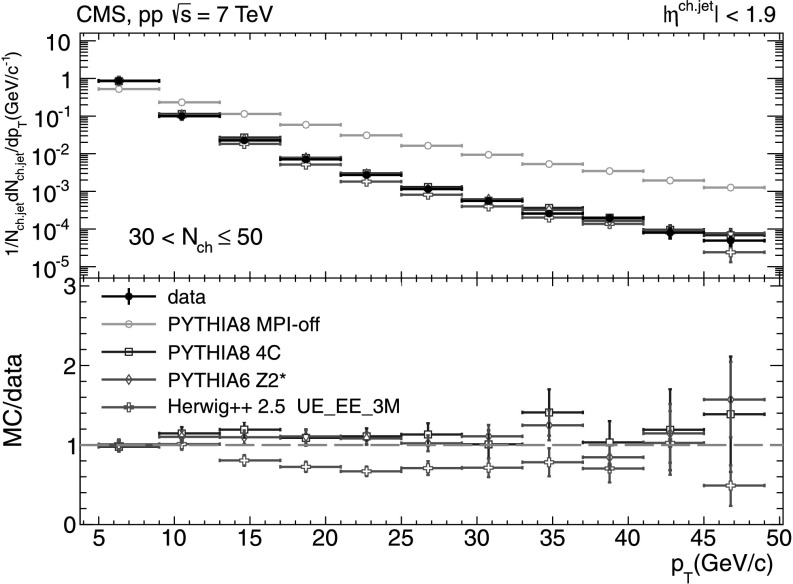
Inclusive charged-particle jet *p*_T_ spectrum for events with 30<*N*_ch_(|*η*|<2.4)≤50 measured in the data (*solid dots*) compared to various MC predictions (*empty markers*). *Error bars* denote the total uncertainties

**Fig. 11:**
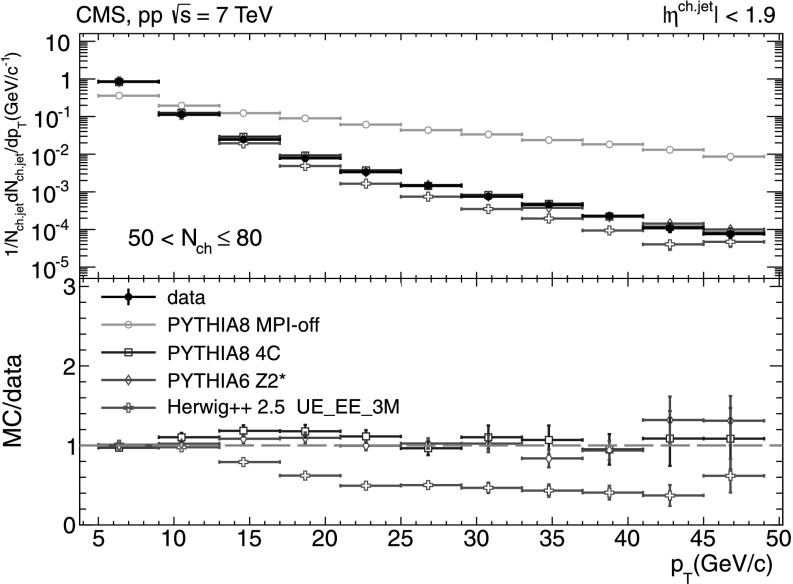
Inclusive charged-particle jet *p*_T_ spectrum for events with 50<*N*_ch_(|*η*|<2.4)≤80 measured in the data (*solid dots*) compared to various MC predictions (*empty markers*). *Error bars* denote the total uncertainties

**Fig. 12:**
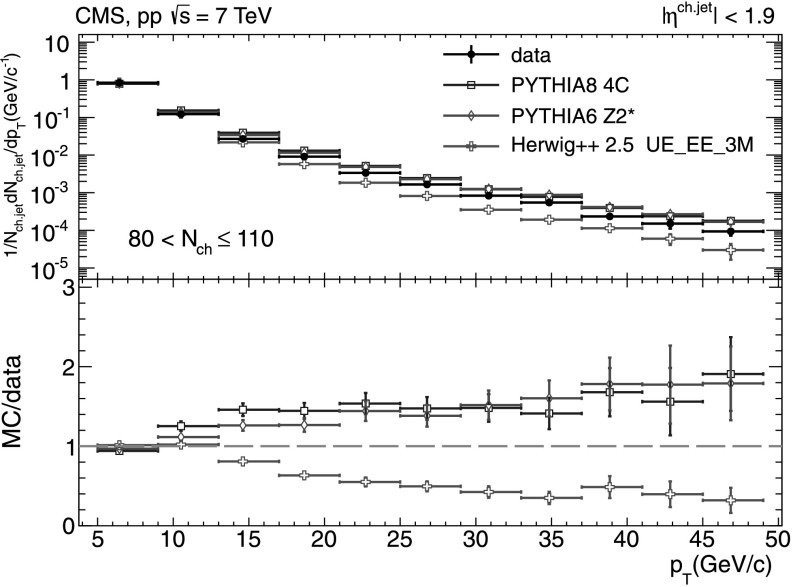
Inclusive charged-particle jet *p*_T_ spectrum for events with 80<*N*_ch_(|*η*|<2.4)≤110 measured in the data (*solid dots*) compared to various MC predictions (*empty markers*). *Error bars* denote the total uncertainties

**Fig. 13:**
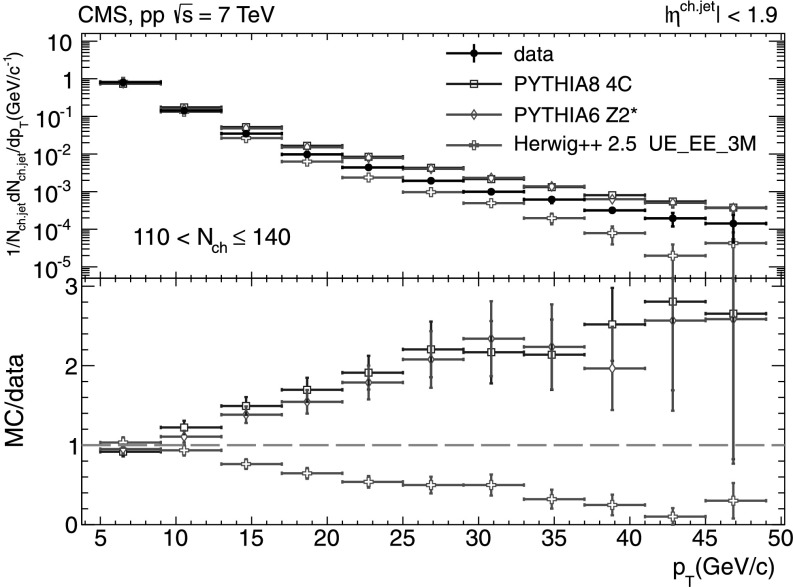
Inclusive charged-particle jet *p*_T_ spectrum for events with 110<*N*_ch_(|*η*|<2.4)≤140 measured in the data (*solid dots*) compared to various MC predictions (*empty markers*). *Error bars* denote the total uncertainties

A more detailed picture of the properties of jet spectra both in data and MC simulations is provided by directly comparing the *p*_T_-differential distributions in each of the five multiplicity bins shown in Figs. 9, 10, 11, 12, 13. In the first three *N*_ch_ bins the measured jet *p*_T_ spectra are reasonably well reproduced by the MC predictions. However, in the two highest-multiplicity bins, 80<*N*_ch_≤110 (Fig. 12) and 110<*N*_ch_≤140 (Fig. 13), we observe much softer jet spectra for transverse momenta *p*_T_>20 GeV/*c* , where data are lower by a factor of ∼2 with respect to pythia predictions. At the same time, herwig++ 2.5 shows the opposite trend, and predicts softer charged-particle jets than measured in data in all multiplicity bins. The relative “softening” of the measured jet spectra compared to pythia at high-*N*_ch_, explains also the higher sphericity of high-multiplicity events observed in Ref. [15].

#### Charged-particle jet widths

**Fig. 14:**
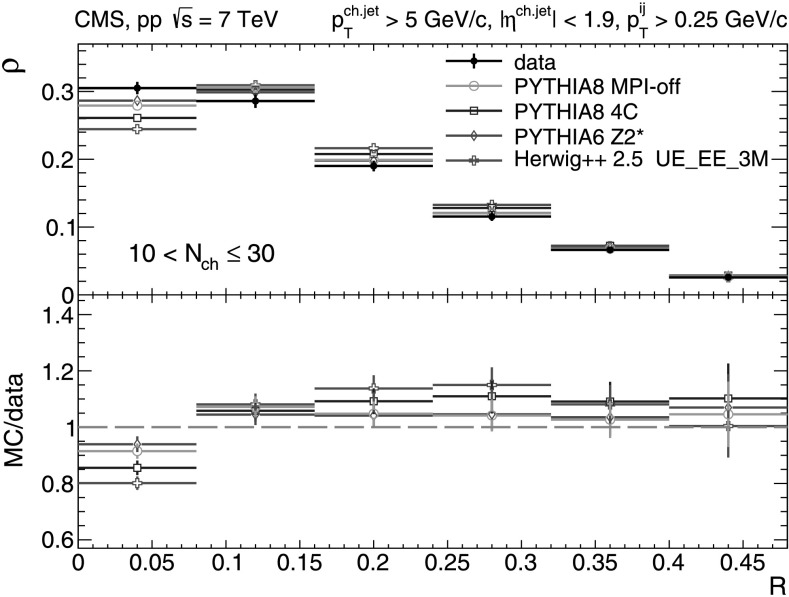
Normalized charged-particle jet *p*_T_ density *ρ* in ring zones as a function of distance to the jet axis *R* for events with 10<*N*_ch_(|*η*|<2.4)≤30 measured in the data (*solid dots*) compared to various MC predictions (*empty markers*). *Error bars* denote the total uncertainties

**Fig. 15:**
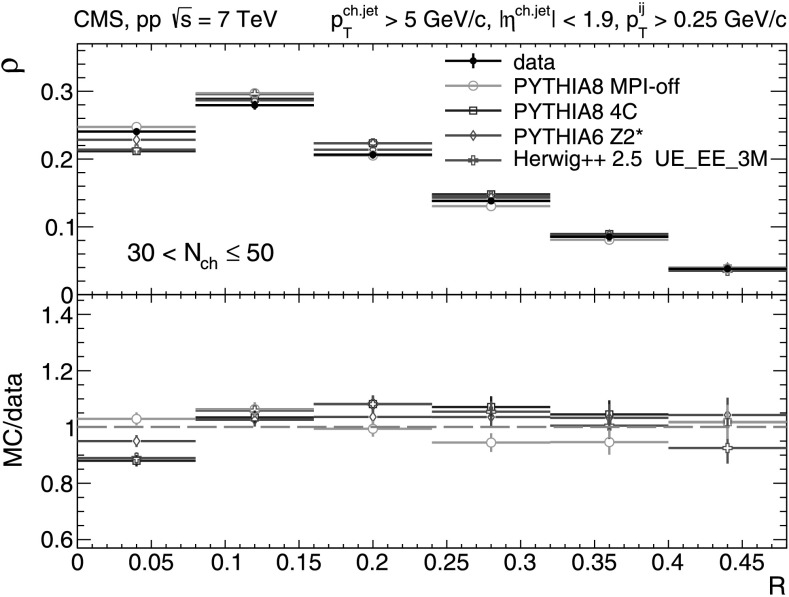
Normalized charged-particle jet *p*_T_ density *ρ* in ring zones as a function of distance to the jet axis *R* for events with 30<*N*_ch_(|*η*|<2.4)≤50 measured in the data (*solid dots*) compared to various MC predictions (*empty markers*). *Error bars* denote the total uncertainties

**Fig. 16:**
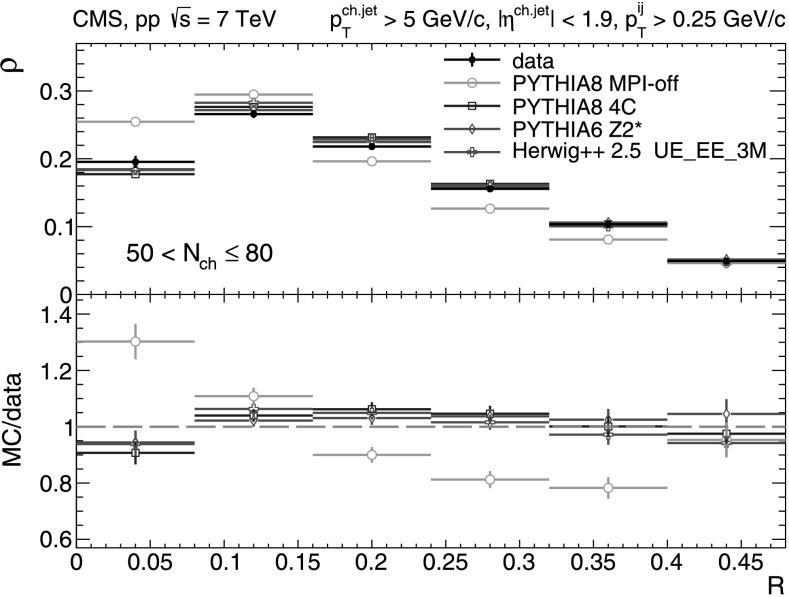
Normalized charged-particle jet *p*_T_ density *ρ* in ring zones as a function of distance to the jet axis *R* for events with 50<*N*_ch_(|*η*|<2.4)≤80 measured in the data (*solid dots*) compared to various MC predictions (*empty markers*). *Error bars* denote the total uncertainties

**Fig. 17:**
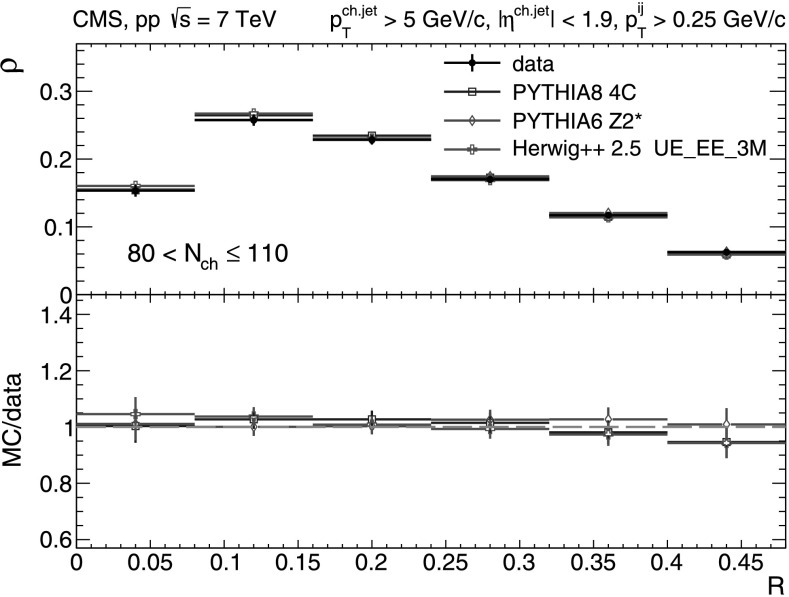
Normalized charged-particle jet *p*_T_ density *ρ* in ring zones as a function of distance to the jet axis *R* for events with 80<*N*_ch_(|*η*|<2.4)≤110 measured in the data (*solid dots*) compared to various MC predictions (*empty markers*). *Error bars* denote the total uncertainties

**Fig. 18:**
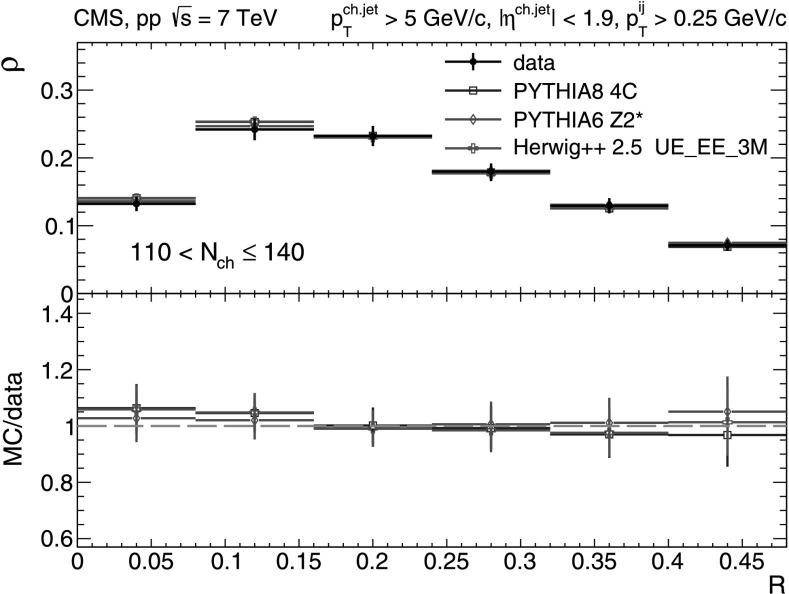
Normalized charged-particle jet *p*_T_ density *ρ* in ring zones as a function of distance to the jet axis *R* for events with 110<*N*_ch_(|*η*|<2.4)≤140 measured in the data (*solid dots*) compared to various MC predictions (*empty markers*). *Error bars* denote the total uncertainties

The jet width provides important information for characterizing the internal jet radiation dynamics. In this analysis, we quantitatively study the jet width through the *p*_T_ charged-particle density in ring zones with respect to the jet center, defined as:

3 $$ \rho= \biggl\langle \frac{1}{p_{\mathrm{T}}^\text{ch. jet}} \frac {\delta p_{\mathrm{T}} ^\text{ch. particles}}{\delta R} \biggr\rangle _\text{ch. jets}, $$

where $R = \sqrt{(\phi-\phi_{\text{jet}})^{2}+(\eta-\eta_{\text{jet}})^{2}}$ is the distance of each charged particle from the jet axis. Larger values of *ρ*(*R*) denote a larger transverse momentum fraction in a particular annulus. Jets with $p_{\mathrm{T}}^{\text{ch. jet}}\geq5~\text{GeV/}c$ are selected for the study. Data are compared with MC predictions in five multiplicity intervals as shown in Figs. 14–18. The dependencies shown in Figs. 14–18 indicate that the jet width increases with *N*_ch_, which can be partly explained by the larger contribution of the UE to jets when *N*_ch_ increases and partly by softer, consequently larger-angle, hadronization, which follows from the intrinsic bias introduced by the requirement of very large values of *N*_ch_. In low-multiplicity events, jets are narrower than predicted by pythia and herwig, whereas in high-multiplicity events they are of comparable width as predicted by the MC event generators. For events with 10<*N*_ch_≤50, the pythia 8 model with MPI switched-off shows jet widths that are close to the ones predicted by the models that include MPI, but it produces too hard jets, which are very collimated, in the bin 50<*N*_ch_≤80. The patterns observed in the data show that the models need to be readjusted to reproduce the activity in the innermost ring zone of the jet as a function of event multiplicity.

## Conclusions

The characteristics of particle production in pp collisions at $\sqrt {s}=7\ \mbox{TeV}$ have been presented as a function of the event charged-particle multiplicity (*N*_ch_) by separating the measured charged particles into those belonging to jets and those belonging to the underlying event. Charged particles are measured within the pseudorapidity range |*η*|<2.4 for transverse momenta *p*_T_>0.25 GeV/*c* and charged-particle jets are reconstructed with *p*_T_ >5 GeV/*c* with charged-particle information only. The distributions of jet *p*_T_, average *p*_T_ of UE charged-particles and jets, jet rates, and jet shapes have been studied as functions of *N*_ch_ and compared to the predictions of the pythia and herwig event generators.

The average trends observed in the data are described by the QCD event generators but the quantitative agreement, in particular at the highest multiplicity, is not as good. The mean transverse momentum of inclusive and UE charged-particles and charged-jets, as well as the charged-jet rates, all rise with *N*_ch_ as expected for an increased fraction of (harder) multiple parton scatterings in more central pp collisions resulting in increasingly higher multiplicity. On the other hand, the average *p*_T_ of the intrajet constituents and the leading charged-particle of the jets decrease (logarithmically) with increasing *N*_ch_ as a result of a selection bias: final states with a larger number of hadrons result from (mini)jets which fragment into more, and thus softer, hadrons. The characteristics of the highest multiplicity pp events result from two seemingly opposite trends: a large number of parton interactions with increasingly harder (mini)jets, combined with an overall softer distribution of final-state hadrons.

The detailed features of the *N*_ch_-dependence of the jet and the UE properties differ from the MC predictions. In general, pythia (and in particular pythia 6 tune Z2*) reproduces the data better than herwig for all observables measured. Of special interest is the large difference between the measured jet *p*_T_-differential spectra and the simulation predictions for the highest-multiplicity bins, above *N*_ch_=80. In these bins jets are softer, and less abundant than predicted by pythia, which explains the observed larger event sphericity compared to predictions [15]. The MC models also fail to fully describe the intrajet spectra. The deviation of simulation predictions from the data for the spectra of the leading intrajet particle is small in comparison to the variation between different models and their tunes, but systematic. In low-multiplicity events, jets are narrower than predicted by pythia and herwig, whereas in high-multiplicity events their widths are as predicted by the MC event generators. At the same time, the characteristics of the UE are well reproduced by most of the MC event generators in all the multiplicity bins considered.

The results obtained in this study are of importance both for improving the MC description of the data and for getting a firmer grasp on the fundamental mechanisms of multi-particle production in hadronic collisions at LHC energies. Current event generators tuned to reproduce the inelastic LHC data cannot describe within a single approach the dependence of various quantities on event multiplicity. This is especially true in the high-multiplicity range, where pythia produces many particles because of increased high-*p*_T_ jet contribution and herwig++ seems to contain too many soft-parton scatterings. The results of pythia with MPI switched off, demonstrate that the MPI mechanism is critical for reproducing the measured properties of the jets and UE for moderate and large charged-particle multiplicities. Taken together, the MC predictions globally bracket the data and indicate possible ways for improving the parameter tuning and/or including new model ingredients.
